# Whole-exome sequencing reveals novel genomic signatures and potential therapeutic targets during the progression of rectal neuroendocrine neoplasm

**DOI:** 10.1038/s41419-024-07232-1

**Published:** 2024-11-15

**Authors:** Shi Xu, Zhi Yong Zhai, Ping Zhou, Xiu Fen Xue, Zhao Yu Huang, Xia Xi Li, Gen Hua Yang, Chong Ju Bao, Li Juan You, Xiao Bing Cui, Gui Li Xia, Mei Ping Ou yang, Long Fei Li, Lan Lu, Wei Gong, Xiao Juan Pei, Wei Hu

**Affiliations:** 1 Department of Burn and Plastic Surgery, Shenzhen Longhua District Central Hospital, Shenzhen, Guangdong China; 2 Department of Medical Laboratory, Shenzhen Longhua District Central Hospital, Shenzhen, Guangdong China; 3 Department of Gastroenterology, Shenzhen Hospital, Southern Medical University, Shenzhen, Guangdong China; 4 The Third School of Clinical Medicine, Southern Medical University, Shenzhen, Guangdong China; 5 Department of Pathology, Shenzhen Hospital, Southern Medical University, Shenzhen, Guangdong China; 6 Institute of Chinese Medicine, State Key Laboratory of Research on Bioactivities and Clinical Applications of Medicinal Plants, The Chinese University of Hong Kong, Shatin, N.T., Hong Kong China; 7 Antibiotics Research and Re-evaluation Key Laboratory of Sichuan Province, Sichuan Industrial Institute of Antibiotics, School of Pharmacy, Chengdu University, Chengdu, Sichuan China

**Keywords:** Rectal cancer, Genetics research

## Abstract

Rectal neuroendocrine neoplasms (rNENs) are among the most frequent gastrointestinal neuroendocrine neoplasms and pose a serious challenge for clinical management. The size of the primary neoplasm is considered to be the most important predictor of disease progression, but the genetic alterations that occur during the progression of rNENs remain unknown. Here, we performed a comprehensive whole-exome sequencing study on 54 tumor-normal paired, formalin-fixed paraffin-embedded specimens from patients locally diagnosed with rNENs. Of these, 81.5% (*n* = 44) were classified as small-sized (≤2 cm) rNENs, while the remainder (18.5%, *n* = 10) were classified as large-sized (>2 cm) rNEN samples. Comparative analysis revealed marked disparities in the mutational landscape between small- and large-sized rNEN samples, and between large-sized rNEN samples with or without lymph node metastases. The high-confidence driver genes *RHPN2*, *MUC16*, and *MUC4* were significantly mutated in both small- and large-sized rNEN specimens, whereas mutations in *MAN2A1*, and *BAG2* were only identified in large-sized specimens diagnosed with lymph node metastases. Correspondingly, we observed that the mTOR and MAPK pathways were preferentially enriched in the large-sized rNEN specimens. Signature-based analysis revealed that mutational processes associated with defective DNA base excision repair (SBS30) significantly accumulated in large-sized rNEN samples with lymph node metastases, highlighting the important role of this mutagenic process in promoting rNEN progression. We further found that most rNEN subjects, regardless of tumor size, harbored at least one alteration with targeted therapeutic implications. Taken together, these results elucidate the genetic features associated with tumor size and lymphatic metastasis in rNEN patients, which will deepen our understanding of the genetic changes during rNEN progression and potentially directing improvements in rNEN treatment strategies.

## Introduction

Neuroendocrine neoplasms (NENs) are a spectrum of heterogeneous malignancies characterized by the loss of epithelial tubular gland structures and extensive expression of neuroendocrine markers, including chromogranin A, CD56, synaptophysin and INSM1 [1–4]. NENs can arise from virtually any organ or system in the body but predominantly affect the lung, bronchi, and gastroenteropancreatic system [5, 6]. According to data from the Surveillance, Epidemiology, and End Results (SEER) registry, the distribution of primary tumors differs by ethnicity, with the lung being the most common site in White individuals, and the rectum being the most common in Asian patients [7]. Rectal NENs (rNENs), which account for 12%–27% of all NENs, have exhibited the greatest increase in incidence among all gastroenteropancreatic NENs in recent decades, probably owing to the widespread use of colonoscopy as a screening tool [8]. rNENs are usually characterized as well-differentiated tumors exhibiting a slow growth associated with a good prognosis. However, regional lymph node metastases and distant metastases are present in 16–40% of patients at the moment of diagnosis. In these cases, the mean survival decreases to a minimum of 5 months [8, 9].

Tumor size is considered to be the most important predictor of disease progression in patients with rNENs [10]. Matsuhashi et al. revealed that rNENs sized <10 mm and without lymphovascular invasion could be considered benign and curatively treated by endoscopic resection. Tumors sized between 1 and 2 cm in diameter have a 10%–15% probability of lymph node and distant metastases. However, rNENs sized >2 cm carry a risk of metastasis equivalent to that of rectal adenocarcinomas, with a 64% probability of lymph node metastases and a 45% probability of distant metastases [11]. Likewise, a single-center retrospective study reported that 292 cases of rNENs (diameter ≤2 cm) with endoscopic resection had no recurrence or metastasis after 0.5–5 years’ postoperative follow-up. However, among the remaining 20 cases, which had diameters >2 cm, 8 patients were diagnosed with intrahepatic metastasis, and 4 of these died due to extensive liver metastasis within the next two years [12].

Several whole-genome sequencing (WGS) and whole-exome sequencing (WES) studies have been performed to elucidate the genetic features of NENs [13–16]. However, these studies mostly focused on pancreatic or gastrointestinal NENs, with very limited sample sizes of rNENs in the study cohorts, leading to inadequate understanding of the biology of rNENs. Moreover, in all of the above studies, the colonic NEN and rNEN samples were grouped together, although it is becoming clear that rNENs differ from colonic NENs in malignancy, differentiation, and disease phenotype [8, 17]. To investigate the mutational landscape during the progression of rNENs, we herein performed WES of tumor-normal paired, formalin-fixed paraffin-embedded (FFPE) specimens from 54 patients locally diagnosed with rNENs, 81.5% (44/54) of which were classified as small-sized rNEN samples (≤2 cm), while the rest (18.5%, 10/54) were classified as large-sized rNEN samples (>2 cm). Among them, 5.6% (*n* = 3) of the small-sized rNEN patients and 70% (*n* = 7) of the large-sized rNEN patients had lymph node or distant metastases. By applying a series of well-established bioinformatic algorithms, we were able to profile the genetic alterations, mutational signatures, and copy number aberrations between small- and large-sized rNEN samples, and between large-sized rNEN samples in the absence or presence of lymph node metastases, and further explore their potential clinical significance. Collectively, our findings will deepen our understanding of the genetic features associated with tumor size and lymphatic metastasis during the progression of rNENs and provide a valuable information source for identification of promising biomarkers, therapeutic targets, and prognostic determinants for this disease.

## Materials and methods

### Sample collection

FFPE samples were obtained from 54 enrolled rNEN patients who underwent endoscopic surgery at Shenzhen Hospital, Southern Medical University from December 2015 to April 2023. No statistical methods were used to predetermine sample size. For sample enrollment, the patients that were diagnosed as rNEN with histological confirmed to be rNEN were enrolled, following the inclusion criteria [1]: between 18 and 80 years old [2]; received resection of the primary tumor in Shenzhen Hospital, Southern Medical University [3]; enough tumor sample for sequencing [4]; no prior chemotherapy, radiotherapy, immunotherapy or other anti-tumor treatment at sampling point [5]; no history of other malignancy. Patients diagnosed with colonic NEN were excluded from this study. rNEN was firstly diagnosed by two independent experienced endoscopists. The hematoxylin and eosin-stained tumor sections from each sample were then examined by two independent pathologists to confirm the diagnosis and histopathological features, including tumor size, mitotic count, tumor grade, depth of invasion, lymphatic invasion, and status of the resection margin. According to the diameters of primary tumors, these samples were classified into rNEN-S and rNEN-L groups. FFPE tumor samples were used for whole-exome sequencing with corresponding adjacent normal tissues used as control. Tumor samples with more than 10% of tumor content were eligible for sequencing. Matched adjacent noncancerous samples were defined as tissues with normally histological morphology and containing no tumor cells. All protocols were approved by the Ethic Committee of Southern Medical University (NYSZYYEC20190013), and written informed consent were obtained from all study participants for the use of tissue samples.

### DNA extraction and library construction

For FFPE samples, genomic DNA was extracted from ten tumor or adjacent noncancerous sections of 5 μm using the QIAamp DNA FFPE Tissue Kit (Qiagen, Hilden, Germany) according to the manufacture’s instruction. DNA quantity was assessed by Nanodrop 2000 spectrophotometer and Qubit 2.0 Fluorometer with Quanti-IT dsDNA HS Assay Kit (Thermo Fisher Scientific, MA, USA). At least 10 ng DNA was prepared for sequencing libraries. Library construction was then performed using a custom 53 M length capturing probe (Integrated DNA Technologies, Coralville, IA, USA). Samples with a DNA library concentration ≥20 ng/uL were then subjected to sequencing. Captured libraries were then pair-end sequenced with Geneplus-2000 sequencing platform (Geneplus, Beijing, China), based on MGI DNBSEQ-G400 sequencer (https://en.mgi-tech.com/products/) which utilized DNA nano-ball (DNB) preparation technology and fluorescent signal detection for base calling.

### Data quality control

The original fluorescent image files obtained from T7 platform are transformed to short reads (Raw data) by base calling and these short reads are recorded in FASTQ format, which contains sequence information and corresponding sequencing quality information. Quality control is applied to exclude sequence artifacts, including reads containing adapter contamination, low-quality nucleotides and unrecognizable nucleotides (N), thus, to guarantee the meaningful downstream analysis. The steps of data processing were as follows: (a) Discard a paired reads if either one read contains adapter contamination (>10 nucleotides aligned to the adapter, allowing ≤10% mismatches); (b) Discard a paired reads if more than 5 bases are uncertain in either one read; (c) Discard a paired reads if the proportion of low quality (Phred quality < 5) bases is over 40% in either one read.

### Alignment and somatic mutation calling for genomes

For each patient, valid sequencing data including tumor and matched normal samples were mapped to the reference human genome (GRCh37/hg19) by Burrows-Wheeler Aligner (BWA, version: 0.7.17) software [18] to get the original mapping results stored in BAM format. If one or one paired read(s) were mapped to multiple positions, the strategy adopted by BWA was to choose the most likely placement. If two or more most likely placements presented, BWA picked one randomly. Then, SAMtools [19] (version: 1.9) and GATK (https://github.com/broadinstitute/gatk, version: 4.1.2) were used to sort BAM files and do duplicate marking, local realignment, and base quality recalibration to generate final BAM file for computation of the sequence coverage and depth. Duplicate reads were uninformative and shouldn’t be considered as evidence for variants. BAM files from both tumor and matched normal samples were used to detect somatic SNV and Indel by GATK Mutect 2 (version: 4.1.2), and GATK AllelicCNV (version: 4.1.2) was used to detect somatic CNV. VEP (version: 93.7) is performed to do annotation for VCF (Variant Call Format) file obtained in the previous step. The variant position, variant type, conservative prediction, and other information are obtained at this step through a variety of databases, such as dbSNP, 1000 Genome, esp6500, GnomAD, CADD, HGMD and COSMIC, and so on. Since we are interested in exonic variants, gene transcript annotation databases, such as Consensus CDS, RefSeq, Ensemble and UCSC, are also applied for annotation to determine amino acid alternation. In this step, the databases mainly include GO, KEGG, Reactome, Biocarta, PID, and so on.

### Somatic mutation filtering

Germline variants were filtered from the Genome Aggregation Database (gnomAD). A stringent downstream filter comprised of the following criteria was used to obtain high quality somatic variants: a minimum of 8× coverage; Variant Allele Fraction (VAF) ≥ 5% and at least 5 variant supporting reads in the tumor sample, and VAF < 1% in the non-tumor sample; strand bias % 0.95; After that, mutations in the non-coding regions (30UTR, 50UTR, Intron, gene intergenic etc.) were removed.

### HC-SMG identification

To detect recurrent mutated gene, a multi-tool approach was implemented to provide a comprehensive analysis. We used four computational tools: dNdScv [20], MutSigCV [21], OncodriveCLUST [22] and OncodriveFML [23]. The rational of each tool was described as follow: The dNdScv R package is designed to compare the rate of non-synonymous (dN) to synonymous (dS) mutations to identify genes under positive selection in cancer, and is particularly useful for datasets ranging from a few samples to thousands, taking into account background mutation rates and various genomic features to increase accuracy. MutSigCV is a classical and well-known frequency method that has been cited thousands of times in cancer genomics. It is a statistical method that identifies significantly mutated genes by considering the mutational landscape of a cancer genome, which is effective for whole genome or whole exome sequencing data and is particularly powerful for identifying cancer driver genes in large-scale studies. OncodriveCLUSTL is a sequence-based clustering algorithm to detect significant clustering signals across genomic regions, which is designed to exploit the feature that mutations in cancer genes, especially oncogenes, often cluster in particular positions of the protein. OncodriveFML is designed to analyze the pattern of somatic mutations across tumors in both coding and non-coding genomic regions to identify signals of positive selection. It is capable of identifying driver mutations in protein-coding genes, promoters, untranslated regions, intronic splice regions, and lncRNAs. These tools were run with the default parameters. SMGs identified by ≥2 tools were considered HC-SMGs. Putative driver genes were searched by mapping these HC-SMGs to the COSMIC Cancer Gene Census [24] and DriverDBv4 [25] databases.

### Tumor mutational burden (TMB) and microsatellite instability (MSI) analysis

TMB was defined as the number of non-synonymous somatic mutations (including base substitutions and indels) in the coding region. In order to calculate the TMB, the total number of mutations counted was divided by the size of the coding sequence region of the Agilent SureSelect Human All Exon V6. Msisensor (v0.5) (https://github.com/ding-lab/msisensor) is used for microsatellite instability analysis which counted the number of known microsatellite loci that were altered by somatic insertion or deletion.

### Recurrently mutated pathway analysis

ClusterProfiler (version 3.12.0) [26] was used to analyze the enrichment of mutated SMGs. Mutated genes were compared with the Kyoto Encyclopedia of Genes and Genomes (KEGG) (http://www.genome.ad.jp/kegg/) database to determine the altered pathways. The *p* values of KEGG pathway enrichment were calculated based on hypergeometric distribution with false discovery rate (FDR) correction using the Benjamini and Hochberg method. Representative key signaling pathways with an FDR-corrected *p* value < 0.05 were exhibited for these rNEN samples.

### Mutational signature analysis

Mutation signatures were jointly inferred with the software of MutationalPatterns (https://bioconductor.org/packages/release/bioc/html/MutationalPatterns.html, version: 1.10.0). The 96 mutational vectors (or contexts) generated by somatic SNVs based on six base substitutions (C > A, C > G, C > T, T > A, T > C, and T > G) within 16 possible combinations of neighboring bases for each substitution were used as input data, then rectified by the FFPEsig algorithm to exclude the formalin-induced artefacts [27].

### Somatic copy number alteration (SCNA) analysis

For each tumor, arm-level and focal CNVs were detected and analyzed by GATK (version 4.0) and GISTIC (version 2.0). Genes encompassed by focal CNVs were also inferred using GISTIC (version 2.0). Significant somatic CNVs were analyzed in a group-wise fashion using GISTIC (version 2.0). Somatic CNV events of each sample were also obtained using GISTIC (version 2.0) to reveal recurrent CNV events, where *P* value was calculated by Fisher’s exact test. The sample-specific CNV data were also used to calculate CNV burden of each sample. A burden score was given to each CNV event based on the amplitude of the log2 copy number ratio of the varied region.

### Analysis of putative clinically relevant alterations

Somatic SNV/Indel and CNVs were analyzed by Precision Heuristics for Interpreting the Alteration Landscape (PHIAL) software [28] (version 1.0.R) (https://github.com/ vanallenlab/phial) with default parameters and database.

### Statistical analysis

Statistical analysis and data visualization were performed using R software (v 4.1.2) or Graphpad Prism (version 9.5.0, GraphPad Software, La Jolla, CA, USA). Two-tailed Wilcoxon rank-sum test was used to analyze the mutational frequencies of HC-SMGs and the contributions of mutational signatures between rNEN-S and rNEN-L samples, and between rNEN-L-M and rNEN-L-N samples. The statistical significance of KEGG presented pathways in rNEN samples using the SMGs list were also calculated by two-tailed Wilcoxon rank-sum test. *P* values presented in Figs. S1 and S2 were calculated by Student’s *t*-test. Statistical significance was defined as a two-sided *p* < 0.05.

## Results

### Overview of the clinicopathological characteristics of the included rNEN patients

The study included a total of 54 patients histopathologically diagnosed as having a rNEN, comprising 28 (51.9%) male and 26 (48.1%) female patients with median ages of 47 (quartile [Q]_1_–Q_3_: 39–54) and 50 (Q_1_–Q_3_: 45–60) at diagnosis, respectively. Among the subjects, 81.5% (*n* = 44) had small- or moderate-sized tumors (diameter < 1 cm or 1–2 cm), denoted as rNEN-S, whereas the remainder (18.5%, *n* = 10) were diagnosed with larger sized neoplasms (diameter >2 cm), denoted as rNEN-L. The tumor sizes of the rNEN-L patients were significantly larger than those of their small-sized counterparts (Figure S1A).

Pathologically, the majority of the rNEN-S specimens (98.1%, *n* = 43) were diagnosed as being at the G1 stage according to the latest World Health Organization (WHO) classification [5]. In contrast, most of the rNEN-L specimens (90%, *n* = 9) were classified as being at the G2 or G3 stages. Neuroendocrine carcinoma samples were not included in this study. Consistent with previous reports that tumor size was an important predictor of disease progression [29, 30], we found that only 5.6% (*n* = 3) of the rNEN-S patients had lymph node metastases, whereas 70% (*n* = 7) of the rNEN-L subjects had lymph node or distant metastases, at the time of biopsy. The clinicopathological characteristics of the rNEN subjects were summarized in Table S1. Figure S1B showed representative colonoscopy and hematoxylin and eosin (H&E) images of rNEN samples.

### Identification of high-confidence Significantly Mutated Genes (HC-SMGs) and their frequencies of loss of heterozygosity (LOH)

To investigate the mutational differences between the rNEN-S and rNEN-L populations, we extracted DNA from all 54 FFPE rNEN samples and their corresponding nonneoplastic tissues for WES using paired-end procedures, to a median mean read coverage of 262× (Q_1_–Q_3_: 150–350×) and 90× (Q_1_–Q_3_: 73–119×), respectively (Table S2). After strict data processing (see filter criteria in the “Method” section), we obtained a total of 32,959 single-nucleotide variants (SNVs; median: 83; Q_1_–Q_3_: 24–222), 5279 small insertions and deletions (InDels; median: 30; Q_1_–Q_3_: 9–114), and 114,216 structural variants (SVs; median: 1,130; Q_1_–Q_3_: 619.5–3150) in the rNEN-S samples. Meanwhile, from the rNEN-L population, we obtained 7,093 SNVs (median: 97.5; Q_1_–Q_3_: 67–369), 883 InDels (median: 33.5; Q_1_–Q_3_: 26–74), and 114,216 SVs (median: 580; Q_1_–Q_3_: 421–794).

**Fig. 1:**
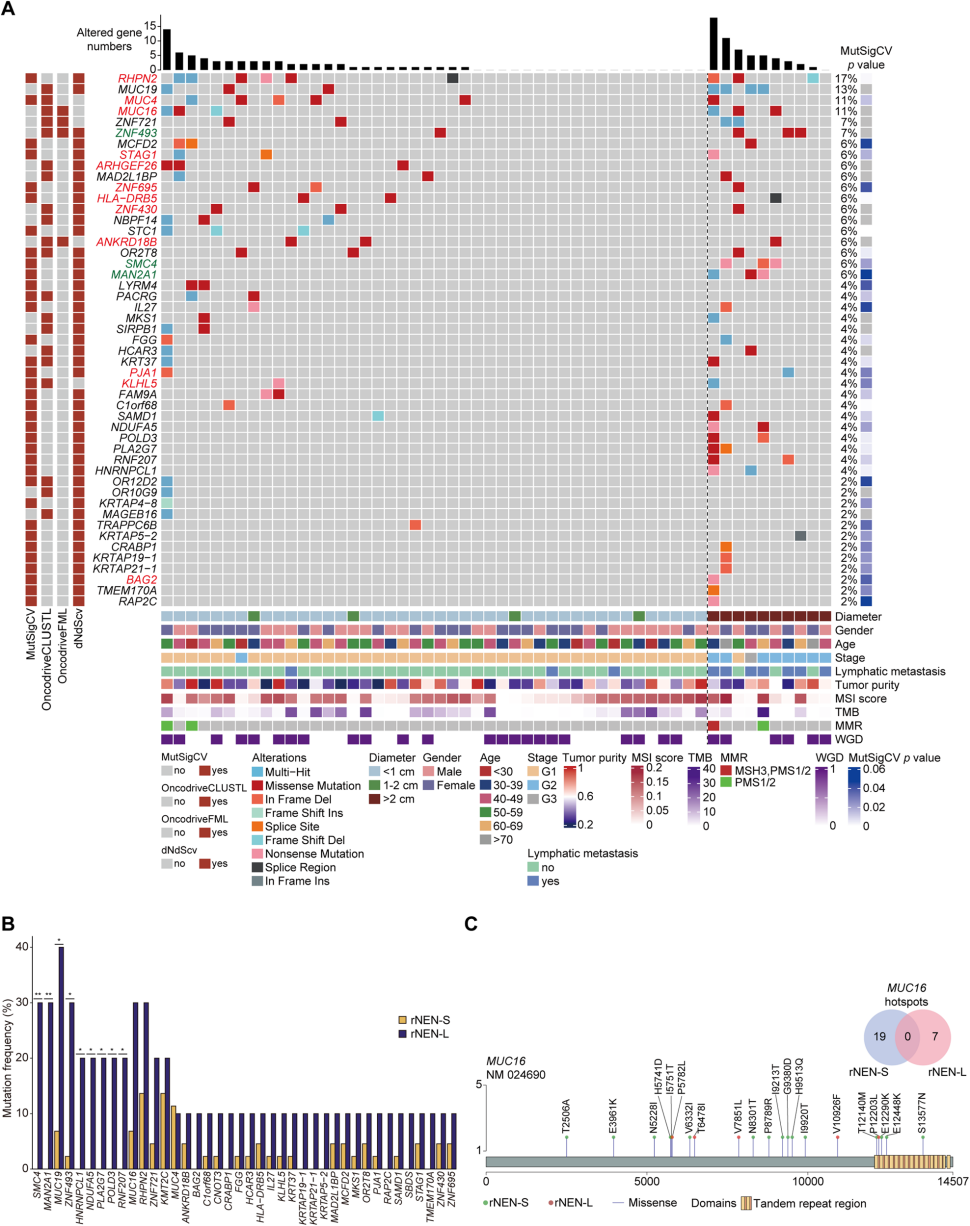
Identification of HC-SMGs in rNENs. **A** SMGs identified by ≥2 algorithms were shown. Driver genes significantly mutated in both rNEN-S and rNEN-L samples were labeled as red color, and driver genes only mutated in rNEN-L specimens were labeled as green color. **Top**: bars represent the number of altered genes in each rNEN sample. **Left**: Algorithms that were used to identify the SMGs. **Right**: Mutation status of the SMGs ranked by mutation frequency in each rNEN sample. *P* values of the SMGs were calculated by the MutSigCV. **Bottom**: The clinicopathological data, MSI scores, TMB, somatic MMR gene mutations, and WGD events are displayed. **B** Mutation frequencies of 40 top HC-SMGs in rNEN-S and rNEN-L samples. *P* values were calculated by Wilcoxon rank sum test. *p < 0.05; **p < 0.01. **C** Lollipop plot showed the distribution of somatic mutations in *MUC16* gene in rNEN-S and rNEN-L samples, respectively. The Ven diagram showed the overlapped hotspots in *MUC16* between rNEN-S and rNEN-L specimens.

Similar to the moderate accumulation of somatic mutations in other NEN cohorts [14], we detected an overall low tumor mutational burden (TMB) in the rNEN-S (median: 0.33 somatic mutations per megabase [Mb], Q_1_–Q_3_: 0.165–1.44) and rNEN-L (median: 0.495 somatic mutations per Mb, Q_1_–Q_3_: 0.18–1.86) specimens (Fig. 1A). No significant difference in TMB was observed between these two cohorts (*p* = 0.918, Wilcoxon rank sum test) (Fig. S2A). MSIsensor was applied to determine the status of microsatellite instability (MSI) in these 54 subjects [31], which revealed that 61.4% of the rNEN-S samples were MSI-high (MSI-H), whereas 40% of the rNEN-L samples were MSI-H (Fig. S2B). As anticipated, we detected significantly higher TMB in the MSI-H samples than in the microsatellite stable (MSS) samples in both the rNEN-S (*p* = 0.04, Wilcoxon rank sum test) and rNEN-L (*p* = 0.014, Wilcoxon rank sum test) populations (Fig. S2C). Somatic mutations in DNA mismatch repair genes were identified in 4.5% and 20% of the rNEN-S and rNEN-L specimens, respectively (Fig. 1A). Whole-genome duplication (WGD) is a macroprogressionary genetic alteration that promotes tumorigenesis by inducing genomic instability [32]. We therefore calculated the frequencies of WGD in these two populations and found that 52.3% (23/44) of the rNEN-S and 60% (6/10) of the rNEN-L samples harbored WGD (Fig. 1A), however, no statistically significant difference was detected between the two groups (*p* = 0.736, Wilcoxon rank sum test). Moreover, we found no significant difference in TMB between the WGD^+^ and WGD^-^ samples in the rNEN-S (*p* = 0.776, Wilcoxon rank sum test) patients or in the rNEN-L (*p* = 0.286, Wilcoxon rank sum test) subjects (Fig. S2D).

To identify genomic alterations that are likely to be linked to tumorigenesis, we applied an integrative screening approach to unbiasedly identify significantly mutated genes (SMGs) using four algorithms, namely MutSigCV (a frequency-based method of estimating highly recurrent mutations within a gene), OncodriveCLUSTL (a sequence-based clustering algorithm for detecting known clusters and bona-fide cancer drivers across genomic regions) [22], OncodriveFML (a method designed to identify signals of positive selection) [23], and dNdScv (a statistical model for predicting variable mutation rates across the genome) [20] (Fig. S3A, Table S3). The number of SMGs identified by each tool was shown in Fig. S3B. Only candidate genes identified by two or more algorithms were considered HC-SMGs. In this way, we obtained 49 HC-SMGs in the 54 rNEN samples (Fig. 1A), among which *RHPN2* (17%), *MUC19* (13%), *MUC4* (11%), *MUC16* (11%), *ZNF721* (7%), *ZNF493* (7%), *MCFD2* (6%), *STAG1* (6%), *ARHGEF26* (6%), *MAD2L1BP* (6%), *ZNF695* (6%), *HLA-DRB5* (6%), *ZNF430* (6%), *NBPF14* (6%), *STC1* (6%), *ANKRD18B* (6%), *OR2T8* (6%), *SMC4* (6%), and *MAN2A1* (6%) exhibited moderate to high mutation prevalence (>5%). Next, we searched for putative driver genes by mapping these HC-SMGs to the COSMIC Cancer Gene Census [24] and DriverDBv4 [25] databases and found that a total of 15 previously known tumor suppressors and oncogenes were enriched in somatic aberrations in these rNEN patients (Fig. 1A). Among them, *MUC16* has been previously reported as a driver gene in pancreatic and midgut-derived NENs [14]. In addition, Duan et al. recently showed that *MUC16* was recurrently mutated in 55.3% of their cohort of rectal NEN patients [33]. The previously unreported putative drivers comprised *RHPN2* (17%), *MUC4* (11%), *ZNF493* (7%), *STAG1* (6%), *ARHGEF26* (6%), *ZNF695* (6%), *HLA-DDRB5* (6%), *ZNF430* (6%), *ANKRD18B* (6%), *SMC4* (6%), *MAN2A1* (6%), *PJA1* (4%), *KLHL5* (4%), and *BAG2* (2%). We also detected several genes enriched in non-synonymous mutations that were previously proposed as driver genes in other NEN cohorts, such as *MEN1*, *KMT2C*, *BPTF*, *ATRX*, and *ARID1*A [13, 14, 16]. However, each of these genes was only identified by one algorithm, so they were not included in our analysis.

Next, we compared the mutational frequencies of these driver genes in rNEN-S and rNEN-L populations and found that *RHPN2*, *MUC16*, and *MUC4*, which were all among the most prevalent in the list of HC-SMGs, were highly mutated in both the rNEN-S (*RHPN2*: 13.64%; *MUC16*: 6.82%, *MUC4*: 11.36%) and rNEN-L (*RHPN2*: 30%; *MUC16*: 30%, *MUC4*: 10%) populations, with no statistically significant difference in their mutation rates between the two populations (*RHPN2*: *p* = 0.06; *MUC16*: *p* = 0.07; *MUC4*: *p* = 0.107; Wilcoxon rank sum test) (Fig. 1B). *RHPN2* (rhophilin Rho GTPase binding protein 2), encoding a member of the rhophilin family of Ras-homologous (Rho)-GTPase binding proteins, is frequently mutated in several cancer types, such as lung cancer [34], hepatocellular carcinoma [35], and ovarian cancer [36]. *MUC16* (mucin 16, cell surface-associated), previously known as carbohydrate antigen 125 (*CA125*), is a mucin marker that is among the most frequently mutated genes in human cancer tissues [37, 38]. *MUC4* (mucin 4, cell surface-associated) is a transmembrane glycoprotein that is implicated in the pathogenesis of various human cancers [39]. Importantly, we found that several driver genes, including *SMC4*, *MAN2A1*, and *ZNF493*, were significantly mutated in the rNEN-L samples (*SMC4*: 30%, *p* = 0.005; *MAN2A1*: 30%, *p* = 0.005; *ZNF493*: 30%, *p* = 0.017; Wilcoxon rank sum test) than in the rNEN-S samples (*SMC4*: 0%; *MAN2A1*: 0%; *ZNF493*: 2.27%) (Fig. 1C), suggesting that these genes were associated with the tumorigenesis or progression of rNEN-L. *SMC4* (structural maintenance of chromosomes 4) is closely related to the occurrence, progression, immune infiltration, and prognosis of various tumor types [40, 41]. *MAN2A1* (mannosidase alpha class 2A member 1) encodes lysosomal alpha-d-mannosidase and is associated with the recurrence of multiple cancers [42]. *ZNF493* (zinc finger protein 493) was reported to be frequently mutated in colorectal cancer [43].

As mentioned above, we detected lymph node or distant metastases in 70% (*n* = 7) of the rNEN-L patients (referred to as rNEN-L-M), while the remainder (30%, *n* = 3) were diagnosed as having no metastatic lymph nodes (referred to as rNEN-L-N) (Fig. 1A, Table S1). To further investigate the mutational discrepancy in these rNEN-L patients with or without lymphatic metastases, we compared the mutation frequencies of these HC-SMGs in rNEN-L-M and rNEN-L-N samples (Fig. S3C), and revealed that HC-SMGs such as *MUC19* (57.1%, *p* = 0.29) and *MAN2A1* (42.9%, *p* = 0.475) were exclusively mutated in rNEN-L-M samples, whereas HC-SMGs, including *ANKRD18B* (33.3%, *p* = 0.3), *ZNF430* (33.3%, *p* = 0.3), *OR2T8* (33.3%, *p* = 0.3), *HLA-DRB5* (33.3%, *p* = 0.3), and *ZNF695* (33.3%, *p* = 0.3) were only mutated in rNEN-L-N specimens. In contrast, we observed that HC-SMGs, including *MUC16* (rNEN-L-M: 14.3%, rNEN-L-N: 66.7%, *p* = 0.18), *SMC4* (rNEN-L-M: 28.6%, rNEN-L-N: 33.3%, *p* = 0.33), *RHPN2* (rNEN-L-M: 28.6%, rNEN-L-N: 33.3%, *p* = 0.33), *ZNF493* (rNEN-L-M: 28.6%, rNEN-L-N: 33.3%, *p* = 0.33), and *ZNF721* (rNEN-L-M: 14.3%, rNEN-L-N: 33.3%, *p* = 0.57) were highly mutated in both rNEN-L-M and rNEN-L-N subjects, with no statistical difference in their mutation rates between the two populations.

We then calculated the frequency of LOH for each HC-SMG. As expected, *RHPN2*, *MUC4*, and *MUC16*, which were among the most highly mutated genes in the total cohort of rNEN samples, had high frequencies of mutation combined with LOH in both the rNEN-S and rNEN-L patients, whereas *SMC4*, *MAN2A1*, and *ZNF493* had high frequencies of LOH only in the rNEN-L subjects (Table S4). Mutational hotspots can help to identify cancer drivers and druggable targets [44]. We therefore explored recurrent point mutations in these rNEN samples using an algorithm developed by Chen et al. [45], by which we identified 46 substitution hotspots in six genes in the rNEN-S specimens and 23 hotspots in four genes in the rNEN-L specimens. Interestingly, we detected a cluster of hotspot mutations distributed in the *MUC16* gene in both the rNEN-S (*n* = 19) and rNEN-L (*n* = 7) samples, while none of these mutations were simultaneously detected in both the populations (Table S5). The loci of individually divergent mutations located in different functional domains of *MUC16*, *MUC4*, *RHPN2*, *SMC4*, and *MAN2A1* in the rNEN-S and rNEN-L populations are presented in Fig. 1C and Fig. S3D.

### Key signaling pathways affected by somatic mutations

**Fig. 2:**
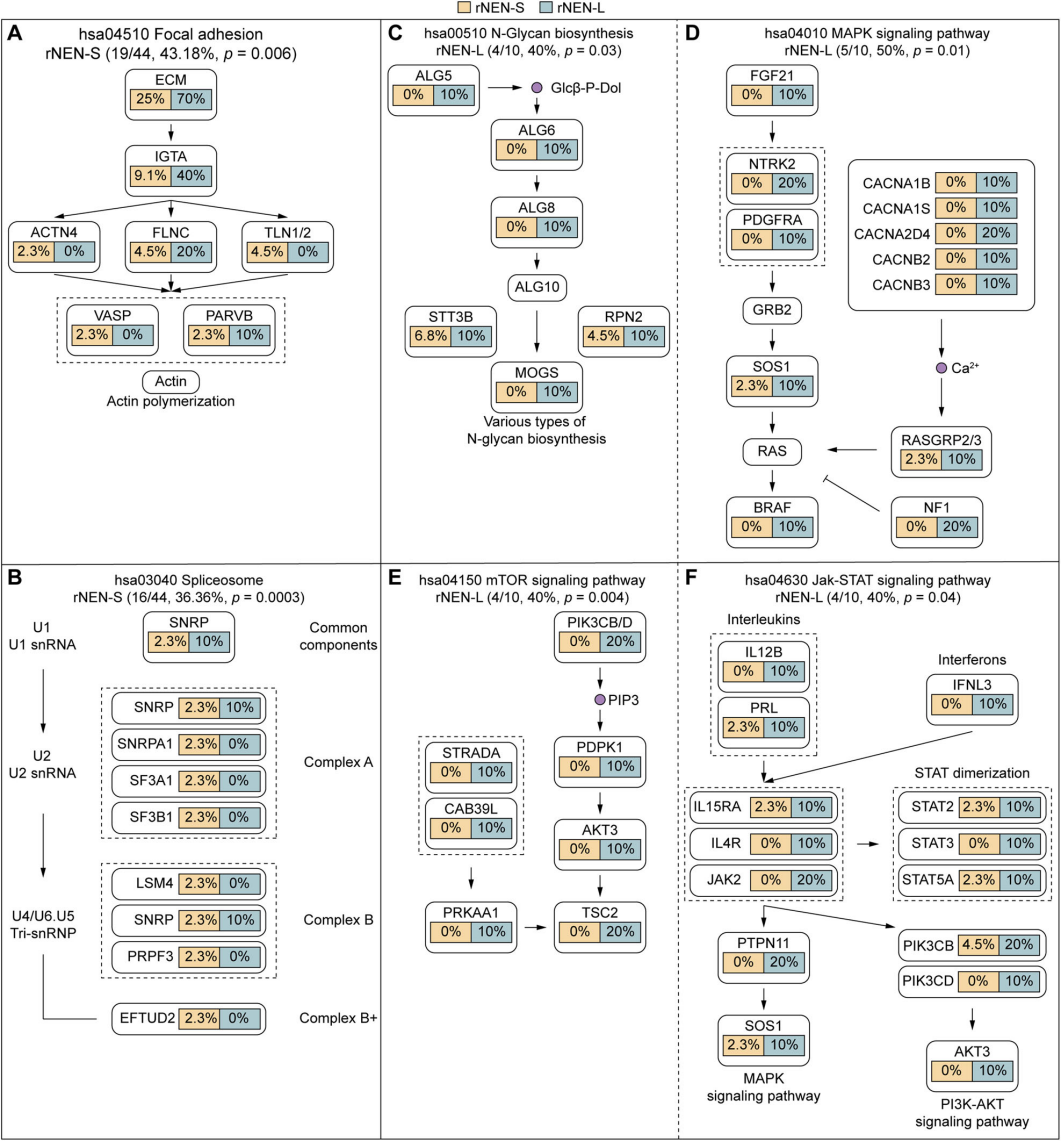
Recurrently mutated key signaling pathways in either rNEN-S or rNEN-L samples. **A**, **B** Focal adhesion (hsa04510) and spliceosome (hsa03040) pathways were recurrently mutated in 43.18% and 36.36% of rNEN-S samples, respectively. **C**–**F** mTOR (hsa04150), N-Glycan biosynthesis (hsa00510), Jak-STAT (hsa04630), and MAPK (hsa04010) signaling pathways were recurrently mutated in 40%, 40%, 40% and 50% of rNEN-L specimens, respectively. Boxes with different colors show the fractions of rNEN-S (**Left**) and rNEN-L (**Right**) samples with alterations in these genes.

Somatic mutations are known to target common cancer signaling pathways. To compare the mutational landscape between the rNEN-S and rNEN-L samples in a pathway-centric manner, we annotated the SMGs identified in these two populations with cancer hallmark pathways. We found that several pathways, including focal adhesion (*p* = 0.006) and spliceosome pathways (*p* = 0.0003), were significantly mutated in 43.18% (19/44) and 36.36% (16/44), respectively, of the rNEN-S samples, while pathways including mTOR (*p* = 0.004), Jak-STAT (*p* = 0.04), MAPK signaling (*p* = 0.01), and N-glycan biosynthesis (*p* = 0.03) were preferentially enriched in 40% (4/10), 40% (4/10), 50% (5/10), and 40% (4/10), respectively, of the rNEN-L specimens (Fig. 2).

We further mapped SMGs identified in rNEN-L-M and rNEN-L-N populations to cell signaling pathways, and revealed that several key biological processes affected by somatic mutations, including citrate cycle (*p* = 0.009), mTOR (*p* = 0.009), N-Glycan biosynthesis (*p* = 0.02), and insulin signaling pathway (*p* = 0.0009) were selectively altered in 42.9% (3/7), 57.1% (4/7), 57.1% (4/7), and 85.7% (6/7), respectively, of the rNEN-L-M samples (Fig. S4).

### Mutational signatures corrected by the FFPEsig algorithm

**Fig. 3:**
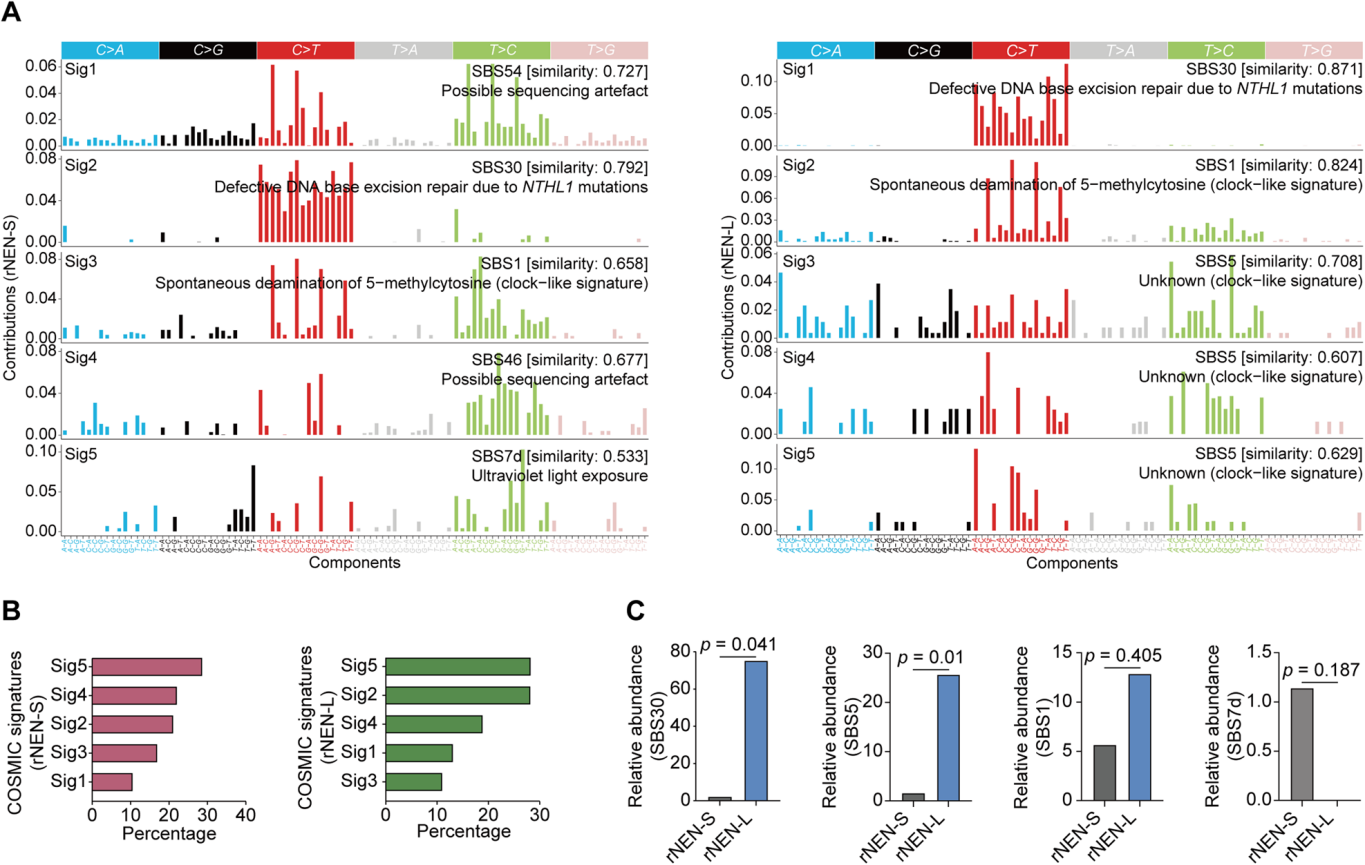
Corrected mutational signatures in rNENs. **A** Mutational signatures were extracted by decomposing matrix of base substitutions, and then rectified by the FFPEsig algorithm. The most similar validated signatures in rNEN-S and rNEN-L samples were shown and cosine-similarities were calculated to identify best match. **B** Bar plots showed the identified mutational signatures and their relative contributions to somatic mutations detected in either rNEN-S or rNEN-L samples. **C** Contribution of SBS30, SBS5, SBS1, and SBS7d in rNEN-S and rNEN-L samples were shown. *P* values were calculated by Wilcoxon rank sum test. *p < 0.05; **p < 0.01.

To better understand the mutational processes operative in the rNEN-S and rNEN-L patients, we performed de novo mutational signature analysis using the SigProfilerExtractor tool to extract the proportions of the 96 possible trinucleotides previously described in Alexandrov’s mutation classification (COSMIC v3) [46]. We further rectified the formalin-induced artefacts in these FFPE-derived DNA samples using the FFPEsig algorithm [27] (Fig. S5A). Finally, we obtained five base-substitution signatures in the rNEN-S and rNEN-L subjects that may contribute to rNEN mutagenesis, denoted as Signature 1 to Signature 5 (Figure S5B). All of these signatures were strongly correlated with previously known mutational signatures (Fig. 3A). In the rNEN-S samples, the main etiology of mutagenesis was Signature 5 (an ultraviolet light exposure-related signature, SBS7d), presenting in 54.5% of the specimens. Signature 4 (associated with possible sequencing artefact, SBS46), Signature 2 (associated with defective DNA base excision repair due to *NTHL1* mutations, SBS30), Signature 3 (associated with spontaneous deamination of 5−methylcytosine, SBS1), and Signature 1 (associated with possible sequencing artefact, SBS54) were present in 45.5%, 52.3%, 38.6%, and 31.8% of the patients, respectively (Fig. 3B). In contrast, in the rNEN-L patients, we detected Signatures 3–5 (associated with SBS5, a flat signature related to the age of individuals) [47], Signature 2 (associated with spontaneous deamination of 5-methylcytosine, SBS1), and Signature 1 (associated with defective DNA base excision repair due to *NTHL1* mutations, SBS30) in 70%, 40%, and 20% of the patients, respectively (Fig. 3B).

SBS1, caused by the deamination of 5­methylcytosine, was simultaneously detected in the rNEN-S (contributing 5.69% of total mutations) and rNEN-L (contributing 12.9% of total mutations) samples, while there was no statistically significant difference in its occurrences between the two populations (*p* = 0.405, Wilcoxon rank sum test) (Fig. 3C). However, we identified that SBS30, one of the dominant signatures, contributed 2.34% and 75.4% of mutations in the rNEN-S and rNEN-L populations, respectively. Significantly higher SBS30 activity in the rNEN-L samples were detected than in the rNEN-S samples (*p* = 0.041, Wilcoxon rank sum test). Likewise, a higher presence of SBS5 (contributing 25.7% of total mutations) in the rNEN-L patients was observed than in the rNEN-S patients (contributing 1.66% of total mutations, *p* = 0.01, Wilcoxon rank sum test). Thus, these results suggest that defective DNA mismatch repair and aging were involved in mutagenesis in the rNEN-L patients.

We further extracted mutational signatures from rNEN-L-M and rNEN-L-N samples, and corrected them using the FFPEsig algorithm (Fig. S6A). As a result, five base-substitution signatures designated as Signature 1 to Signature 5 were profiled in rNEN-L-M and rNEN-L-N specimens, respectively (Fig. S6B). In rNEN-L-M samples, we detected Signature 1 (associated with SBS30), Signature 2 (associated with SBS1), and Signature 3-5 (associated with SBS5) in 14.3%, and 28.6%, and 57.1% of the subjects, respectively. In contrast, Signature 1 (associated with SBS1), Signature 2 (associated with defective DNA mismatch repair, SBS6), Signature 3 (associated with defective DNA mismatch repair, SBS15), and Signature 4&5 (associated with thiopurine chemotherapy, SBS87) were identified in 33.3%, 33.3%, 33.3%, and 33.3% of the rNEN-L-N patients, respectively (Fig. S6C, D). We also examined the contributions of several predominant signatures in rNEN-L-M and rNEN-L-N populations, and revealed increased SBS30 (*p* = 0.66, Wilcoxon rank sum test) and SBS1 (*p* = 0.73, Wilcoxon rank sum test) activities in rNEN-L-M samples compared to their rNEN-L-N counterparts. On the contrary, higher SBS15 (*p* = 0.09, Wilcoxon rank sum test) and SBS87 (*p* = 0.06, Wilcoxon rank sum test) activities were preferentially enriched in rNEN-L-N subjects (Fig. S6E).

### Somatic copy number aberration (SCNAs)

**Fig. 4:**
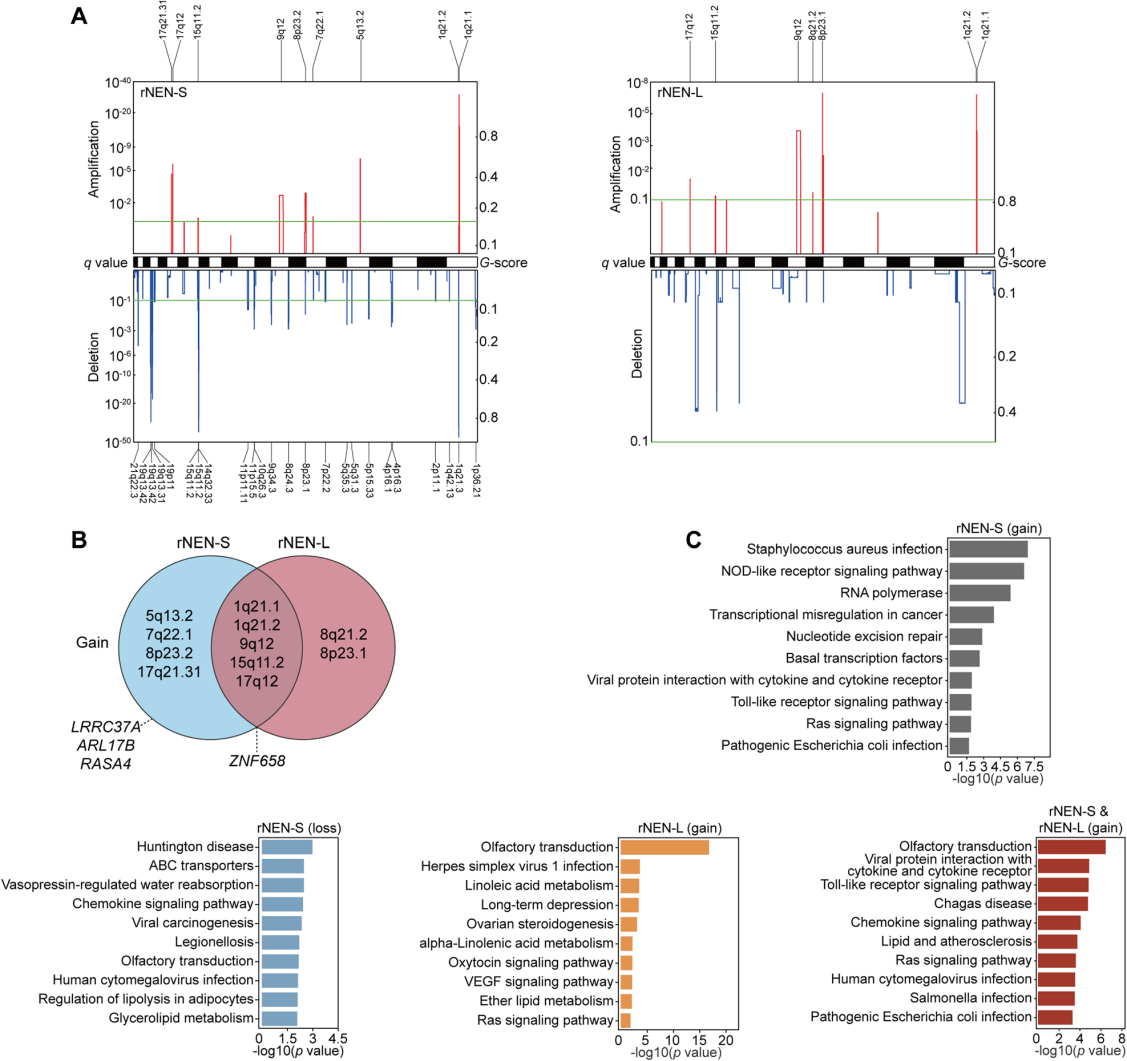
Landscape of significantly altered somatic CNVs in rNENs. **A** Genomic regions with significantly recurrent somatic CNVs in either rNEN-S or rNEN-L samples. **B** Overlap of focal amplification regions detected in rNEN-S and rNEN-L subjects. **C** Pathway enrichment analysis showed the top 10 pathways using genes with a copy number gain or lost in rNEN-S and rNEN-L samples.

Next, we applied the GISTIC2.0 tool to identify the recurrent SCNAs that might contribute to tumorigenesis in the rNEN-S and rNEN-L populations (Fig. 4A). In this way, we found a total of 31 focal events, including 6101.6 Mb copy number gains and 3255.8 Mb copy number losses involving 177 amplified and 1492 deleted genes, across the rNEN-S cohort. In contrast, seven focal amplifications, including 3734.4 Mb gains involving 107 amplified genes, were identified in the rNEN-L patients. We did not detect any significant focal deletions in the rNEN-L samples. Five focal amplifications, namely 1q21.1, 1q21.2, 9q12, 15q11.2, and 17q12, were simultaneously captured in both the cohorts. We further pinpointed the driver SCNAs by mapping genes with copy number aberrations to the DriverDBv4 database [25] and identified three genes, namely *LRRC37A*, *ARL17B*, and *RASA4* with copy number gains were driver SCNAs (Fig. 4B). These genes were exclusively detected in the rNEN-S subjects. *ZNF658* (Zinc finger protein 658), with a copy number gain, annotated as a potential driver SCNA, was detected in both the rNEN-S and rNEN-L populations.

To further understand the biological function of the genes with copy number aberrations, we searched for over-represented pathways in the Kyoto Encyclopedia of Genes and Genomes (KEGG) database using the list of genes with recurrent SCNAs in rNEN-S or rNEN-L subjects. The top 10 altered signaling pathways (*q* < 0.05, Wilcoxon rank sum test) are shown in Fig. 4C. Pathways related to nucleotide excision repair and Toll-like receptor were selectively enriched using genes with a copy number gain in the rNEN-S cohort. Likewise, we observed that ABC transporters, chemokine signaling and glycerolipid metabolism-related pathways were specifically presented using genes with a copy number loss in the rNEN-S subjects. Functional enrichment of genes with a copy number gain revealed that pathways related to linoleic acid metabolism, VEGF signaling, and ether lipid metabolism were markedly enriched in the rNEN-L cohort. Furthermore, we showed that Ras and chemokine-associated pathways were simultaneously presented in rNEN-S and rNEN-L samples using the amplificated gene shared by these two populations.

We then analyzed recurrent SCNV events in rNEN-L-M and rNEN-L-N populations and identified 6 focal amplifications involving 73 genes in rNEN-L-M subjects and 2 focal amplifications involving 12 genes in rNEN-L-N samples (Fig. S7A). The 8p23.1 focal amplification event was identified simultaneously in both populations (Fig. S7B). Functional enrichment analysis of genes with copy number gain showed that several pathways, including olfactory transduction and nitrogen metabolism, were specifically enriched in rNEN-L-M samples. In contrast, Toll-like receptor- and chemokine-related pathways were selectively enriched in rNEN-L-N samples (Fig. S7C). Thus, these results showed that SCNVs in rNEN-L-M subjects had similarities and differences with those in rNEN-L-N patients.

### Therapeutic implications of somatic mutations

**Fig. 5:**
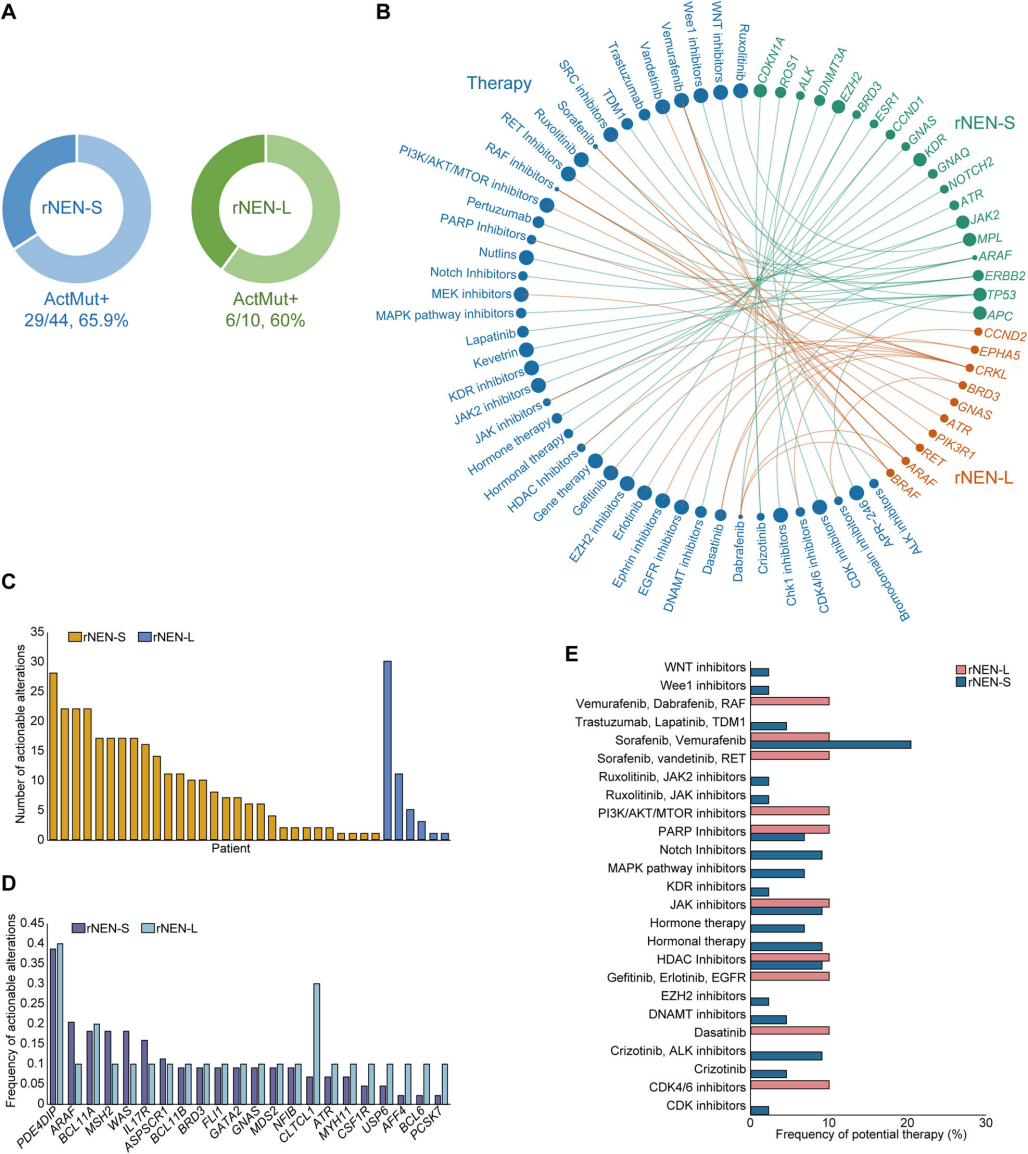
Clinically relevant somatic alterations in rNENs. **A** Proportions of patients harboring clinically relevant somatic alterations in either rNEN-S or rNEN-L samples. ActMut+ indicates patients with somatic actionable alterations. **B** Landscape of somatic altered genes and their corresponding putative therapeutic implications in either rNEN-S or rNEN-L samples. Colors of the circles indicate disease groups; sizes of the circles stand for the frequencies of clinically relevant somatic alterations in these genes or their corresponding putative therapeutic implications. **C** Numbers of clinically relevant somatic alterations in either rNEN-S or rNEN-L samples. **D** Frequencies of clinically relevant somatic alterations in commonly altered genes in either rNEN-S or rNEN-L samples. **E** Proportions of patients that might benefit from or resist to specific therapies in either rNEN-S or rNEN-L subjects.

We next used the Precision Heuristics for Interpreting the Alteration Landscape (PHIAL) database to assess the therapeutic implications of the somatic alterations profiled in these rNEN subjects [48] and found that a total of 65.9% (29/44) of rNEN-S patients and 60% (6/10) of rNEN-L patients had at least one clinically relevant genetic alteration (Fig. 5A). Putatively actionable alterations that could be targeted, together with possible therapies, are shown in Fig. 5B. The number of potentially actionable changes varied between the rNEN-S (median: 9, Q_1_–Q_3_: 2–17) and rNEN-L (median: 4.5, Q_1_–Q_3_: 1–10) patients (Fig. 5C). The therapeutic implications also differed between the two populations. *PDE4DIP* was identified as the most common putatively actionable alteration in both the rNEN-S and rNEN-L populations, followed by *ARAF*, *BCL11A*, *MSH2*, *WAS*, and *IL17R* (Fig. 5D). Compared with the rNEN-S patients, we detected a higher frequency of actionable alterations of *CLTCL1* in the rNEN-L patients. Correspondingly, we inferred that rNEN-L patients may benefit from or be resistant to PI3K/AKT/mTOR inhibitors, gefitinib, dasatinib, and CDK4/6 inhibitors, whereas therapeutic implications of sorafenib, PARP, JAK, and HDAC inhibitors were identified in both rNEN-S and rNEN-L patients (Fig. 5E).

Similarly, 71.4% (5/7) of rNEN-L-M patients and 33.3% (1/3) of rNEN-L-N patients were found to have at least one clinically relevant genetic alteration (Fig. S8A). Putative actionable alterations with their indicated therapies are shown in Fig. S8B. The number of actionable alterations varied between rNEN-L-M (median: 5, Q1–Q3: 3–11) and rNEN-L (median: 1) patients (Fig. S8C). We also identified different therapeutic implications in these two populations (Fig. S8D, E). Compared to rNEN-L-N patients, we identified a higher frequency of actionable alterations in *PDE4DIP*, *CLTCL1*, *BCL11A*, and *TET2* in rNEN-L-M patients, with corresponding therapeutic implications for RET, PI3K/AKT/MTOR and JAK inhibitors, and so on. In contrast, *CRKL* was identified as having a higher frequency of putative actionable alterations in rNEN-L-N patients, with therapeutic implications for EGFR inhibitors in these subjects.

## Discussion

rNENs are the most common digestive NENs in several countries and ethnic groups, and their incidence has increased substantially over recent decades [6]. However, the incidence and prevalence of this disease are still underestimated, due to the frequent failure to recognize it by endoscopy. Several predictors of disease progression, metastatic spread, and poor prognosis of rNENs have been proposed, among which the diameter of the primary neoplasm is the most important [10]. However, the genetic differences between small-sized, benign rNENs and their large-sized counterparts remain poorly understood. In this context, we herein performed a comprehensive and in-depth WES study to dissect the genetic diversity between small- and large-sized rNEN samples, and between large-sized rNEN samples in the absence or presence of lymph node metastases. Comparative analysis revealed marked disparities in their mutational landscapes during the progression of this neoplasm.

In this study, we adopted an integrative screening approach, mainly referenced from a recent publication on colorectal cancer genomics [49], to identify HC-SMGs that are likely to be involved in the tumorigenesis of rNENs. HC-SMGs, i.e., SMGs identified by multiple bioinformatics algorithms rather than a single algorithm, can be prioritized for further investigation. In this way, 49 HC-SMGs in these rNEN samples were detected by two or more algorithms, including some, such as *MUC16*, that were also discovered by previous NEN studies [14, 33]. Notably, several SMGs profiled in our cohort, such as *RHPN2* and *MUC4*, had never been previously reported, likely due to the large sample size of rNEN patients in this study compared with previously studied NEN cohorts. This discovery implies that the genomic alteration signatures in rNENs are markedly distinct from those in other types of NEN. *RHPN2*, which was mutated in 13.64% and 30% of our rNEN-S and rNEN-L subjects, respectively, is required for the proliferation and invasion of multiple cancer cells [34] and can be used as a prognostic biomarker for patients with surgically resected colorectal cancer [50]. *MUC4*, mutated in 11.36% and 10% of our rNEN-S and rNEN-L subjects, has also been revealed to be aberrantly expressed in a variety of epithelial carcinomas and can serve as a clinical tool for cancer diagnosis and prognosis [51]. We further identified that putative driver genes enriched in non-synonymous mutations, including *ZNF493*, *SMC4*, and *MAN2A1*, were substantially mutated in the rNEN-L specimens, but not in the rNEN specimens. Moreover, HC-SMGs such as *MUC19*, *MAN2A1* and *HNRNPCL1* were exclusively mutated in rNEN-L patients with lymph node metastases, suggesting their involvement in the increasingly aggressive behavior of this disease. However, further in vitro and in vivo investigation of their functions in rNENs is necessary.

To reveal the underlying exogenous or endogenous mutagens driving rNEN tumorigenesis, we characterized and compared the landscapes of mutational processes in small- and large-sized rNEN samples, and large-sized rNEN samples with or without lymph node metastases. These deconvoluted mutational signatures were further rectified using the FFPEsig algorithm to remove artefactual FFPE mutations [27]. We showed that endogenous mutational processes, including defective DNA base excision repair (SBS30) and spontaneous deamination of 5-methylcytosine (SBS1), were enriched in the majority of samples in both the rNEN-S and rNEN-L populations. However, unlike SBS1, which contributed similarly in both the populations, SBS30 was significantly more frequent in the rNEN-L specimens, particularly in rNEN-L samples in the presence of metastatic lymph nodes, suggesting an important role of this mutagenic process in promoting rNEN progression. Likewise, we also found that SBS5, a clock-like signature mutation that increases in number with age, was significantly more frequent in the rNEN-L samples than in the rNEN-S samples, implying that age is a risk factor for rNEN progression. Indeed, the age at diagnosis was higher in the rNEN-L cohort than in the rNEN-S cohort, although this difference was not statistically significant (*p* = 0.07, unpaired Student’s *t* test), likely due to the relatively small sample size of the rNEN-L cohort.

At the level of SCNV events, we observed several focal amplifications, including 1q21.1, 1q21.2, 9q12, 15q11.2, and 17q12 in both small- and large-sized rNEN samples, whereas the focal amplifications 8q21.2 and 8q23.1 were detected exclusively in rNEN-L patients. By annotating genes with copy number aberrations in the DriverDBv4 database, we were able to identify several genes with high frequency and important SCNVs, including *LRRC37A*, *ARL17B* and *RASA4*, in rNEN-S patients. In contrast, amplification of *ZNF658* on 9q12, a potential driver SCNA, was detected in both rNEN-S and lymph node metastatic rNEN-L subjects. Interestingly, pathways related to viral infection were significantly enriched in rNEN-S and rNEN-L patients with or without lymph node metastases, which is reminiscent of numerous publications suggesting the involvement of viral infection in the development of neuroendocrine neoplasms [52–54].

Currently, the choice of therapeutic strategy for rNENs is largely dependent on whether the tumors are resectable or not. For intramucosal rNENs with a diameter <1 cm, endoscopic procedures are considered sufficient and usually associated with desirable long-term results. However, for locally advanced, unresectable neoplasms, pharmacological treatment, including biotherapy and chemotherapy, prevails. Several clinical trials have shown that somatostatin analogues, mTOR inhibitors (everolimus), and VEGF pathway inhibitors (sunitinib) have efficacy in patients with GI or lung NENs [55, 56]. In this study, using the PHIAL database, we revealed that most of the rNEN subjects, regardless of their tumor size and lymph node metastases, harbored at least one alteration with targeted therapeutic implications. In line with the results of previous clinical trials, we also showed that both rNEN-S and rNEN-L patients may benefit from VEGF inhibitor therapy (sorafenib) and the feasibility of EGFR inhibitors (gefitinib) and CDK4/6 inhibitors in the treatment of rNEN-L patients. In particular, our findings suggest that rNEN-L patients diagnosed with lymph node metastases may be more sensitive to RET inhibitors (sorafenib, vandetinib) as well as PI3K/AKT/MTOR and JAK inhibitors. However, their effectiveness should be further assessed in future clinical investigations.

In conclusion, this study has identified several genetic features associated with tumor size and lymphatic metastasis in rNEN patients, which will deepen our understanding of the genetic alterations that occur during rNEN progression and potentially directing improvements in rNEN treatment strategies.

## Supplementary information

Supplemental materials

Table S1

Table S2

Table S3

Table S4

Table S5

## Data Availability

The raw sequence data reported in this paper have been deposited in the Genome Sequence Archive (Genomics, Proteomics & Bioinformatics 2021) in National Genomics Data Center (Nucleic Acids Res 2022), China National Center for Bioinformation/Beijing Institute of Genomics, Chinese Academy of Sciences (GSA-Human: HRA006369) that are publicly accessible at https://ngdc.cncb.ac.cn/gsa-human.

## References

[1] Caplin ME, Ratnayake GM. Diagnostic and therapeutic advances in neuroendocrine tumours. Nat Rev Endocrinol. 2021;17:81–2. 33335329 10.1038/s41574-020-00458-x

[2] Kawasaki K, Fujii M, Sato T. Gastroenteropancreatic neuroendocrine neoplasms: genes, therapies and models. Dis Model Mech. 2018;11. 10.1242/dmm.029595 PMC5894937 29590641

[3] Wiedenmann B, Franke WW, Kuhn C, Moll R, Gould VE. Synaptophysin: a marker protein for neuroendocrine cells and neoplasms. Proc Natl Acad Sci USA. 1986;83:3500–4. 3010302 10.1073/pnas.83.10.3500 PMC323544

[4] Mahalakshmi B, Baskaran R, Shanmugavadivu M, Nguyen NT, Velmurugan BK. Insulinoma-associated protein 1 (INSM1): a potential biomarker and therapeutic target for neuroendocrine tumors. Cell Oncol (Dordr). 2020;43:367–76. 32219703 10.1007/s13402-020-00505-9 PMC12990676

[5] Nagtegaal ID, Odze RD, Klimstra D, Paradis V, Rugge M, Schirmacher P, et al. The 2019 WHO classification of tumours of the digestive system. Histopathology. 2020;76:182–8. 31433515 10.1111/his.13975 PMC7003895

[6] Cives M, Strosberg JR. Gastroenteropancreatic neuroendocrine tumors. CA Cancer J Clin. 2018;68:471–87. 30295930 10.3322/caac.21493

[7] Yao JC, Hassan M, Phan A, Dagohoy C, Leary C, Mares JE, et al. One hundred years after “carcinoid”: epidemiology of and prognostic factors for neuroendocrine tumors in 35,825 cases in the United States. J Clin Oncol. 2008;26:3063–72. 18565894 10.1200/JCO.2007.15.4377

[8] Starzynska T, Deptala A, Krolicki L, Kunikowska J, Londzin-Olesik M, Nasierowska-Guttmejer A, et al. Colorectal neuroendocrine neoplasms - management guidelines (recommended by the Polish Network of Neuroendocrine Tumours). Endokrynol Pol. 2013;64:494–504. 24431120 10.5603/EP.2013.0032

[9] Bertani E, Ravizza D, Milione M, Massironi S, Grana CM, Zerini D, et al. Neuroendocrine neoplasms of rectum: a management update. Cancer Treat Rev. 2018;66:45–55. 29684743 10.1016/j.ctrv.2018.04.003

[10] Gallo C, Rossi RE, Cavalcoli F, Barbaro F, Boskoski I, Invernizzi P, et al. Rectal neuroendocrine tumors: current advances in management, treatment, and surveillance. World J Gastroenterol. 2022;28:1123–38. 35431507 10.3748/wjg.v28.i11.1123 PMC8985485

[11] Matsuhashi N, Takahashi T, Tomita H, Araki H, Ibuka T, Tanaka K, et al. Evaluation of treatment for rectal neuroendocrine tumors sized under 20 mm in comparison with the WHO 2010 guidelines. Mol Clin Oncol. 2017;7:476–80. 28894583 10.3892/mco.2017.1326 PMC5582451

[12] Yangong H, Shi C, Shahbaz M, Zhengchuan N, Wang J, Liang B, et al. Diagnosis and treatment experience of rectal carcinoid (a report of 312 cases). Int J Surg. 2014;12:408–11. 24631555 10.1016/j.ijsu.2014.03.002

[13] Puccini A, Poorman K, Salem ME, Soldato D, Seeber A, Goldberg RM, et al. Comprehensive genomic profiling of gastroenteropancreatic neuroendocrine neoplasms (GEP-NENs). Clin Cancer Res. 2020;26:5943–51. 32883742 10.1158/1078-0432.CCR-20-1804 PMC8970533

[14] van Riet J, van de Werken HJG, Cuppen E, Eskens F, Tesselaar M, van Veenendaal LM, et al. The genomic landscape of 85 advanced neuroendocrine neoplasms reveals subtype-heterogeneity and potential therapeutic targets. Nat Commun. 2021;12:4612. 34326338 10.1038/s41467-021-24812-3 PMC8322054

[15] Banck MS, Kanwar R, Kulkarni AA, Boora GK, Metge F, Kipp BR, et al. The genomic landscape of small intestine neuroendocrine tumors. J Clin Invest. 2013;123:2502–8. 23676460 10.1172/JCI67963 PMC3668835

[16] Scarpa A, Chang DK, Nones K, Corbo V, Patch AM, Bailey P, et al. Whole-genome landscape of pancreatic neuroendocrine tumours. Nature. 2017;543:65–71. 28199314 10.1038/nature21063

[17] Ramage JK, De Herder WW, Delle Fave G, Ferolla P, Ferone D, Ito T, et al. ENETS consensus guidelines update for colorectal neuroendocrine neoplasms. Neuroendocrinology. 2016;103:139–43. 26730835 10.1159/000443166

[18] Li H, Durbin R. Fast and accurate short read alignment with Burrows-Wheeler transform. Bioinformatics. 2009;25:1754–60. 19451168 10.1093/bioinformatics/btp324 PMC2705234

[19] Li H, Handsaker B, Wysoker A, Fennell T, Ruan J, Homer N, et al. The Sequence Alignment/Map format and SAMtools. Bioinformatics. 2009;25:2078–9. 19505943 10.1093/bioinformatics/btp352 PMC2723002

[20] Martincorena I, Raine KM, Gerstung M, Dawson KJ, Haase K, Van Loo P, et al. Universal patterns of selection in cancer and somatic tissues. Cell. 2017;171:1029–41.e21. 29056346 10.1016/j.cell.2017.09.042 PMC5720395

[21] Lawrence MS, Stojanov P, Polak P, Kryukov GV, Cibulskis K, Sivachenko A, et al. Mutational heterogeneity in cancer and the search for new cancer-associated genes. Nature. 2013;499:214–8. 23770567 10.1038/nature12213 PMC3919509

[22] Arnedo-Pac C, Mularoni L, Muinos F, Gonzalez-Perez A, Lopez-Bigas N. OncodriveCLUSTL: a sequence-based clustering method to identify cancer drivers. Bioinformatics. 2019;35:4788–90. 31228182 10.1093/bioinformatics/btz501 PMC6853674

[23] Mularoni L, Sabarinathan R, Deu-Pons J, Gonzalez-Perez A, Lopez-Bigas N. OncodriveFML: a general framework to identify coding and non-coding regions with cancer driver mutations. Genome Biol. 2016;17:128. 27311963 10.1186/s13059-016-0994-0 PMC4910259

[24] Sondka Z, Bamford S, Cole CG, Ward SA, Dunham I, Forbes SA. The COSMIC Cancer Gene Census: describing genetic dysfunction across all human cancers. Nat Rev Cancer. 2018;18:696–705. 30293088 10.1038/s41568-018-0060-1 PMC6450507

[25] Liu CH, Lai YL, Shen PC, Liu HC, Tsai MH, Wang YD, et al. DriverDBv4: a multi-omics integration database for cancer driver gene research. Nucleic Acids Res. 2024;52:D1246–D52. 37956338 10.1093/nar/gkad1060 PMC10767848

[26] Yu G, Wang LG, Han Y, He QY. clusterProfiler: an R package for comparing biological themes among gene clusters. OMICS. 2012;16:284–7. 22455463 10.1089/omi.2011.0118 PMC3339379

[27] Guo Q, Lakatos E, Bakir IA, Curtius K, Graham TA, Mustonen V. The mutational signatures of formalin fixation on the human genome. Nat Commun. 2022;13:4487. 36068219 10.1038/s41467-022-32041-5 PMC9448750

[28] Van Allen EM, Wagle N, Stojanov P, Perrin DL, Cibulskis K, Marlow S, et al. Whole-exome sequencing and clinical interpretation of formalin-fixed, paraffin-embedded tumor samples to guide precision cancer medicine. Nat Med. 2014;20:682–8. 24836576 10.1038/nm.3559 PMC4048335

[29] Chan DT, Luk AO, So WY, Kong AP, Chow FC, Ma RC, et al. Natural history and outcome in Chinese patients with gastroenteropancreatic neuroendocrine tumours: - a 17-year retrospective analysis. BMC Endocr Disord. 2016;16:12. 26911576 10.1186/s12902-016-0087-9 PMC4766724

[30] McDermott FD, Heeney A, Courtney D, Mohan H, Winter D. Rectal carcinoids: a systematic review. Surg Endosc. 2014;28:2020–6. 24584484 10.1007/s00464-014-3430-0

[31] Niu B, Ye K, Zhang Q, Lu C, Xie M, McLellan MD, et al. MSIsensor: microsatellite instability detection using paired tumor-normal sequence data. Bioinformatics. 2014;30:1015–6. 24371154 10.1093/bioinformatics/btt755 PMC3967115

[32] Prasad K, Bloomfield M, Levi H, Keuper K, Bernhard SV, Baudoin NC, et al. Whole-genome duplication shapes the aneuploidy landscape of human cancers. Cancer Res. 2022;82:1736–52. 35502547 10.1158/0008-5472.CAN-21-2065 PMC9069772

[33] Duan X, Zhao M, Yin X, Mi L, Shi J, Li N, et al. Molecular typing and mutational characterization of rectal neuroendocrine neoplasms. Cancer Med. 2023;12:16207–20. 37387515 10.1002/cam4.6281 PMC10469650

[34] Xiao D, He J, Guo Z, He H, Yang S, Huang L, et al. Rhophilin-2 upregulates glutamine synthetase by stabilizing c-Myc protein and confers resistance to glutamine deprivation in lung cancer. Front Oncol. 2020;10:571384. 33552953 10.3389/fonc.2020.571384 PMC7855701

[35] Yuan B, Bo W, Feng X, Hu Y, Zeng J. Overexpression of rhophilin Rho GTPase-binding protein 2 promotes hepatocellular carcinoma. Oncol Lett. 2020;20:382. 33154780 10.3892/ol.2020.12245 PMC7608026

[36] Yu F, Qiao P, Yin G, Sun Y, Yu X, Sun X, et al. RHPN2 promotes malignant cell behaviours in ovarian cancer by activating STAT3 signalling. Onco Targets Ther. 2020;13:11517–27. 33204106 10.2147/OTT.S272752 PMC7667185

[37] Kim N, Hong Y, Kwon D, Yoon S. Somatic mutaome profile in human cancer tissues. Genomics Inform. 2013;11:239–44. 24465236 10.5808/GI.2013.11.4.239 PMC3897852

[38] Aithal A, Rauth S, Kshirsagar P, Shah A, Lakshmanan I, Junker WM, et al. MUC16 as a novel target for cancer therapy. Expert Opin Ther Targets. 2018;22:675–86. 29999426 10.1080/14728222.2018.1498845 PMC6300140

[39] Carraway KL, Theodoropoulos G, Kozloski GA, Carothers Carraway CA. Muc4/MUC4 functions and regulation in cancer. Fut Oncol. 2009;5:1631–40. 10.2217/fon.09.125 PMC2825673 20001800

[40] Jiang G, Chen J, Li Y, Zhou J, Wang W, Wu G, et al. Association of SMC4 with prognosis and immune infiltration of sarcoma. Aging (Albany NY). 2023;15:567–82. 36719264 10.18632/aging.204503 PMC9925680

[41] Zhao Z, Wang X, Ding Y, Cao X, Zhang X. SMC4, a novel tumor prognostic marker and potential tumor therapeutic target. Front Oncol. 2023;13:1117642. 37007153 10.3389/fonc.2023.1117642 PMC10064883

[42] Wang Y, Zhao J, Fu G, Sheng C, Zhu J, Zhong T, et al. MAN2A1 predicts prognosis and progression through cancer-related pathways in colorectal cancer. Transl Cancer Res. 2022;11:3686–97. 36388015 10.21037/tcr-22-629 PMC9641108

[43] Liang Y, Jiang L, Zhong X, Hochwald SN, Wang Y, Huang L, et al. Discovery of aberrant alteration of genome in colorectal cancer by exome sequencing. Am J Med Sci. 2019;358:340–9. 31445671 10.1016/j.amjms.2019.07.012

[44] Vogelstein B, Papadopoulos N, Velculescu VE, Zhou S, Diaz LA Jr, Kinzler KW. Cancer genome landscapes. Science. 2013;339:1546–58. 23539594 10.1126/science.1235122 PMC3749880

[45] Chen T, Wang Z, Zhou W, Chong Z, Meric-Bernstam F, Mills GB, et al. Hotspot mutations delineating diverse mutational signatures and biological utilities across cancer types. BMC Genomics. 2016;17:394. 27356755 10.1186/s12864-016-2727-x PMC4928158

[46] Alexandrov LB, Kim J, Haradhvala NJ, Huang MN, Tian Ng AW, Wu Y, et al. The repertoire of mutational signatures in human cancer. Nature. 2020;578:94–101. 32025018 10.1038/s41586-020-1943-3 PMC7054213

[47] Koh G, Degasperi A, Zou X, Momen S, Nik-Zainal S. Mutational signatures: emerging concepts, caveats and clinical applications. Nat Rev Cancer. 2021;21:619–37. 34316057 10.1038/s41568-021-00377-7

[48] Bailey MH, Tokheim C, Porta-Pardo E, Sengupta S, Bertrand D, Weerasinghe A, et al. Comprehensive characterization of cancer driver genes and mutations. Cell. 2018;173:371–85.e18. 29625053 10.1016/j.cell.2018.02.060 PMC6029450

[49] Zhao Q, Wang F, Chen YX, Chen S, Yao YC, Zeng ZL, et al. Comprehensive profiling of 1015 patients’ exomes reveals genomic-clinical associations in colorectal cancer. Nat Commun. 2022;13:2342. 35487942 10.1038/s41467-022-30062-8 PMC9055073

[50] Kang BW, Jeon HS, Chae YS, Lee SJ, Park JY, Choi JE, et al. Association between GWAS-identified genetic variations and disease prognosis for patients with colorectal cancer. PLoS ONE. 2015;10:e0119649. 25799222 10.1371/journal.pone.0119649 PMC4370892

[51] Singh AP, Chaturvedi P, Batra SK. Emerging roles of MUC4 in cancer: a novel target for diagnosis and therapy. Cancer Res. 2007;67:433–6. 17234748 10.1158/0008-5472.CAN-06-3114

[52] Banerjee J, Ranjan RP, Alam MT, Deshmukh S, Tripathi PP, Gandhi S, et al. Virus-associated neuroendocrine cancers: Pathogenesis and current therapeutics. Pathol Res Pract. 2023;248:154720. 37542862 10.1016/j.prp.2023.154720

[53] Masunaga A, Inoue K, Mizukami H, Hayashi T, Mitsuya T. Neuroendocrine carcinoma arising in a hepatitis C virus-infected liver: mechanism of the tumor development may be similar to that of development of pancreatic neuroendocrine cells. Pathol Int. 2014;64:81–5. 24629176 10.1111/pin.12138

[54] Giordano G, Corcione L, Giordano D, D’Adda T, Gnetti L, Ferri T. Primary moderately differentiated neuroendocrine carcinoma (atypical carcinoid) of the larynx: a case report with immunohistochemical and molecular study. Auris Nasus Larynx. 2009;36:228–31. 18617341 10.1016/j.anl.2008.05.002

[55] Yao JC, Fazio N, Singh S, Buzzoni R, Carnaghi C, Wolin E, et al. Everolimus for the treatment of advanced, non-functional neuroendocrine tumours of the lung or gastrointestinal tract (RADIANT-4): a randomised, placebo-controlled, phase 3 study. Lancet. 2016;387:968–77. 26703889 10.1016/S0140-6736(15)00817-X PMC6063317

[56] Caplin ME, Pavel M, Cwikla JB, Phan AT, Raderer M, Sedlackova E, et al. Lanreotide in metastatic enteropancreatic neuroendocrine tumors. N Engl J Med. 2014;371:224–33. 25014687 10.1056/NEJMoa1316158

