# Supplementary material for: Whole-exome sequencing reveals novel genomic signatures and potential therapeutic targets during the progression of rectal neuroendocrine neoplasm
Source: Cell Death Dis. 2024 Nov 15;15(11):833. doi: 10.1038/s41419-024-07232-1 (PMC11568169; doi:10.1038/s41419-024-07232-1)
Supplement: Supplementary file 1 — Supplemental materials [file 41419_2024_7232_MOESM1_ESM.pdf]

Supplemental figure legends

Figure S1. (A) Comparison of tumor sizes bwtween rNEN-S and rNEN-L samples. *P* values were calculated by Student's *t*-test. ***$p < 0.001$. **(B)** Representative endoscopic and H&E images of rNEN-S and rNEN-L samples.

Figure S2. (A) Comparison of TMB values between rNEN-S and rNEN-L samples. **(B)** Percentage of MSS, MSI-L and MSI-H samples in rNEN-S and rNEN-L subjects. **(C)** Comparison of TMB values between MSI-H and MSS samples in rNEN-S (left) and rNEN-L (right) cohorts, respectively. **(D)** Comparison of TMB values between WGD^{+} and WGD^{-} samples in rNEN-S (left) and rNEN-L (right) cohorts, respectively. *P* values were calculated by Student's *t*-test. *$p < 0.05$; **$p < 0.01$. Abbreviations: MSS: Microsatellite Stable; MSI-L: Microsatellite Instability-low; MSI-H: Microsatellite Instability-high; WGD: Whole-genome duplication.

Figure S3. (A) Algorithms used to identify HC-SMGs. **(B)** Overlap analysis of the recurrent genes identitied by each algorithm. **(C)** Mutation frequencies of HC-SMGs in rNEN-L-M and rNEN-L-N samples. *P* values were calculated by Wilcoxon rank sum test. **(D)** Lollipop plots showed the distribution of somatic mutations in *MUC4*, *SMC4*, *RHPN2*, *MAN2A1* genes in rNEN-S and rNEN-L samples, respectively.

Figure S4. (A) Citrate cycle (TCA cycle, hsa00020), **(B)** mTOR signaling pathway (hsa04150), **(C)** N-Glycan biosynthesis (hsa00510), and **(D)** Insulin signaling pathway (hsa04910) were recurrently mutated in 42.9%, 57.1%, 57.1% and 85.7%of rNEN-L-M samples, respectively. Boxes with different colors show the fractions of rNEN-L-M (**Left**) and rNEN-L-N (**Right**) samples with alterations in these genes.

Figure S5. (A) Observed distribution of the somatic SNVs across the 96 possible mutation types in rNEN-S and rNEN-L samples. **(B)** Contribution scores of the five mutational signatures in rNEN-S (**left**) and rNEN-L (**right**) samples. Upper panel on the top shows the absolute contribution scores of these signatures in each patient; Down

panel shows the relative contribution of these signatures in each patient.

Figure S6. **(A)** Observed distribution of the somatic SNVs across the 96 possible mutation types in rNEN-L-M and rNEN-L-N samples. **(B)** Contribution scores of the five mutational signatures in rNEN-L-M (**left**) and rNEN-L-N (**right**) samples. **(C)** Mutational signatures were extracted by decomposing matrix of base substitutions, and then rectified by the FFPEsig algorithm. The most similar validated signatures in rNEN-L-M and rNEN-L-N samples were shown and cosine-similarities were calculated to identify best match. **(D)** Bar plots showed the identified mutational signatures and their relative contributions to somatic mutations detected in either rNEN-L-M or rNEN-L-N samples. **(E)** Contribution of SBS30, SBS1, SBS15, and SBS87 in rNEN-L-M and rNEN-L-N samples were shown. *P* values were calculated by Wilcoxon rank sum test.

Figure S7. **(A)** Genomic regions with significantly recurrent somatic CNVs in either rNEN-L-M or rNEN-L-N samples. **(B)** Overlap of focal amplification regions detected in rNEN-L-M and rNEN-L-N subjects. **(C)** Pathway enrichment analysis showed the top 10 pathways using genes with a copy number gain in rNEN-L-M or rNEN-L-N samples.

Figure S8. **(A)** Proportions of patients harboring clinically relevant somatic alterations in either rNEN-L-M or rNEN-L-N samples. ActMut+ indicates patients with somatic actionable alterations. **(B)** Landscape of somatic altered genes and their corresponding putative therapeutic implications in either rNEN-L-M or rNEN-L-N samples. Colors of the circles indicate disease groups; sizes of the circles stand for the frequencies of clinically relevant somatic alterations in these genes or their corresponding putative therapeutic implications. **(C)** Numbers of clinically relevant somatic alterations in either rNEN-L-M or rNEN-L-N samples. **(D)** Frequencies of clinically relevant somatic alterations in commonly altered genes in either rNEN-L-M or rNEN-L-N samples. **(E)** Proportions of patients that might benefit from or resist to specific therapies in either rNEN-L-M or rNEN-L-N subjects.

Table S1. rNEN patients' clinical information

Table S2. Summary of whole-exome sequencing statistics for the rNEN cohort.

Table S3. SMGs identified using four algorithms in the rNEN cohort.

Table S4. Frequency of mutation combined with LOH of each HC-SMG in the rNEN cohort.

Table S5. Mutational hotspots identified in the rNEN cohort.

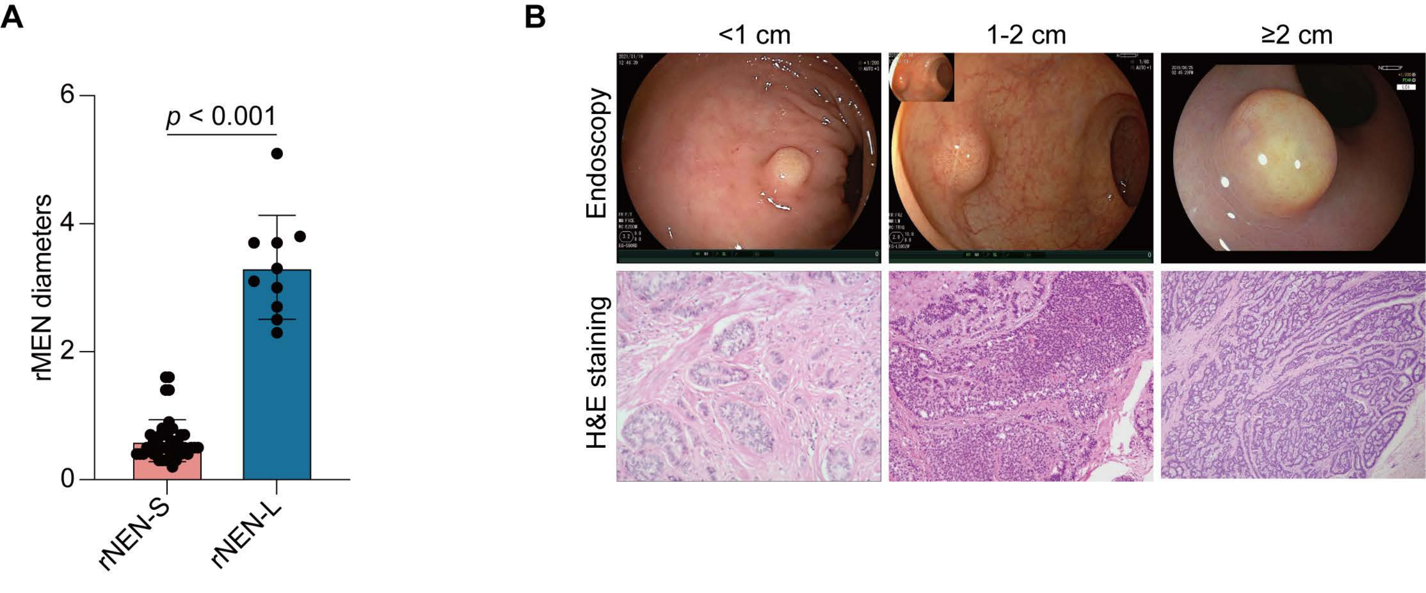
A
B
6
4
2
0
p < 0.001
rMEN diameters
rNEN-S
rNEN-L
<1 cm
1-2 cm
≥2 cm
Endoscopy
H&E staining

Figure S1

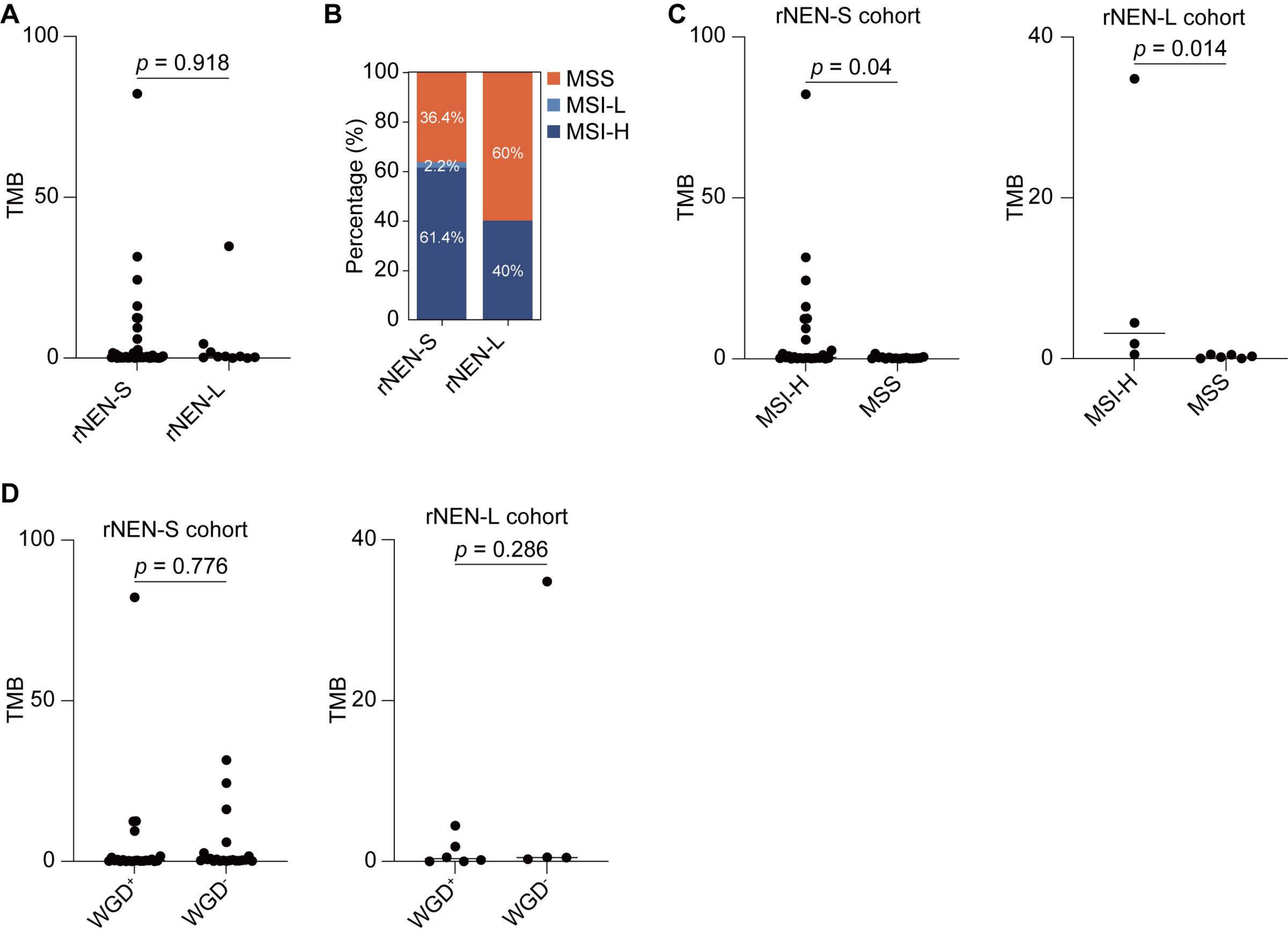

A
B
C
D
TMB
p = 0.918
rNEN-S
rNEN-L
Percentage (%)
MSS
MSI-L
MSI-H
36.4%
2.2%
61.4%
60%
40%
rNEN-S cohort
rNEN-L cohort
p = 0.04
p = 0.014
MSI-H
MSS
p = 0.776
p = 0.286
WGD+
WGD-

Figure S2

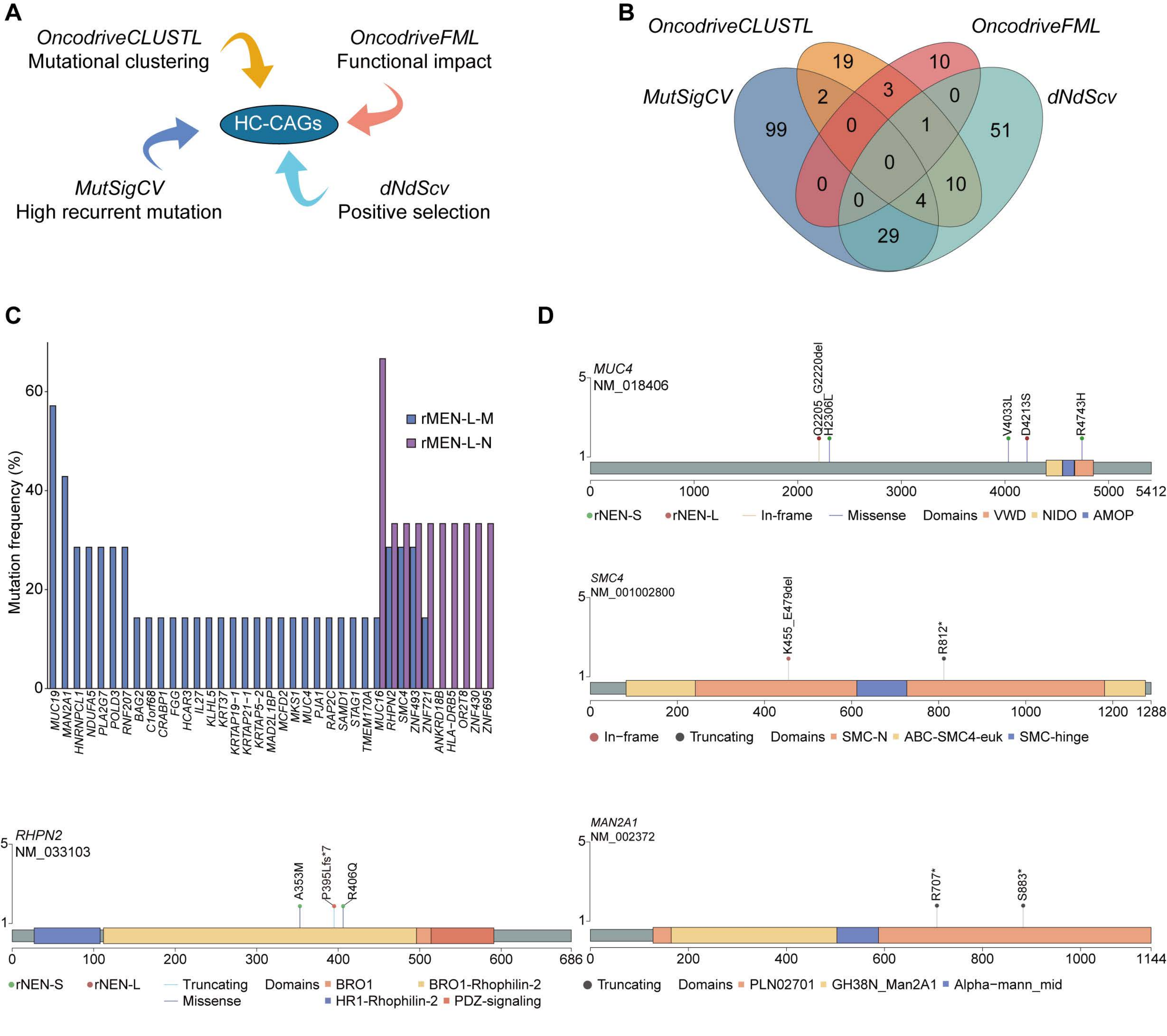

A
OncodriveCLUSTL
Mutational clustering
OncodriveFML
Functional impact
HC-CAGs
MutSigCV
High recurrent mutation
dNdScv
Positive selection
B
OncodriveCLUSTL
OncodriveFML
MutSigCV
dNdScv
19
10
2
3
0
99
0
1
51
0
0
0
10
0
4
29
C
Mutation frequency (%)
rMEN-L-M
rMEN-L-N
MUC19
MAN2A1
HNRNPCL1
NDUFA5
PLA2G7
POLD3
RNF207
BAG2
C1orf68
CRABP1
FGG
HCAR3
IL27
KLHL5
KRT37
KRTAP19-1
KRTAP21-1
KRTAP5-2
MAD2L1BP
MCFD2
MKS1
MUC4
PJA1
RAP2C
SAMD1
STAG1
TMEM170A
MUC16
RHPN2
SMC4
ZNF493
ZNF721
ANKRD18B
HLA-DRB5
OR2T8
ZNF430
ZNF695
D
MUC4
NM_018406
Q2205_G2220del
H2306L
V4033L
D4213S
R4743H
rNEN-S
rNEN-L
In-frame
Missense
Domains
VWD
NIDO
AMOP
SMC4
NM_001002800
K455_E479del
R812*
In−frame
Truncating
Domains
SMC-N
ABC-SMC4-euk
SMC-hinge
RHPN2
NM_033103
A353M
P395Lfs*7
R406Q
rNEN-S
rNEN-L
Truncating
Missense
Domains
BRO1
BRO1-Rhophilin-2
HR1-Rhophilin-2
PDZ-signaling
MAN2A1
NM_002372
R707*
S883*
Truncating
Domains
PLN02701
GH38N_Man2A1
Alpha−mann_mid

Figure S3

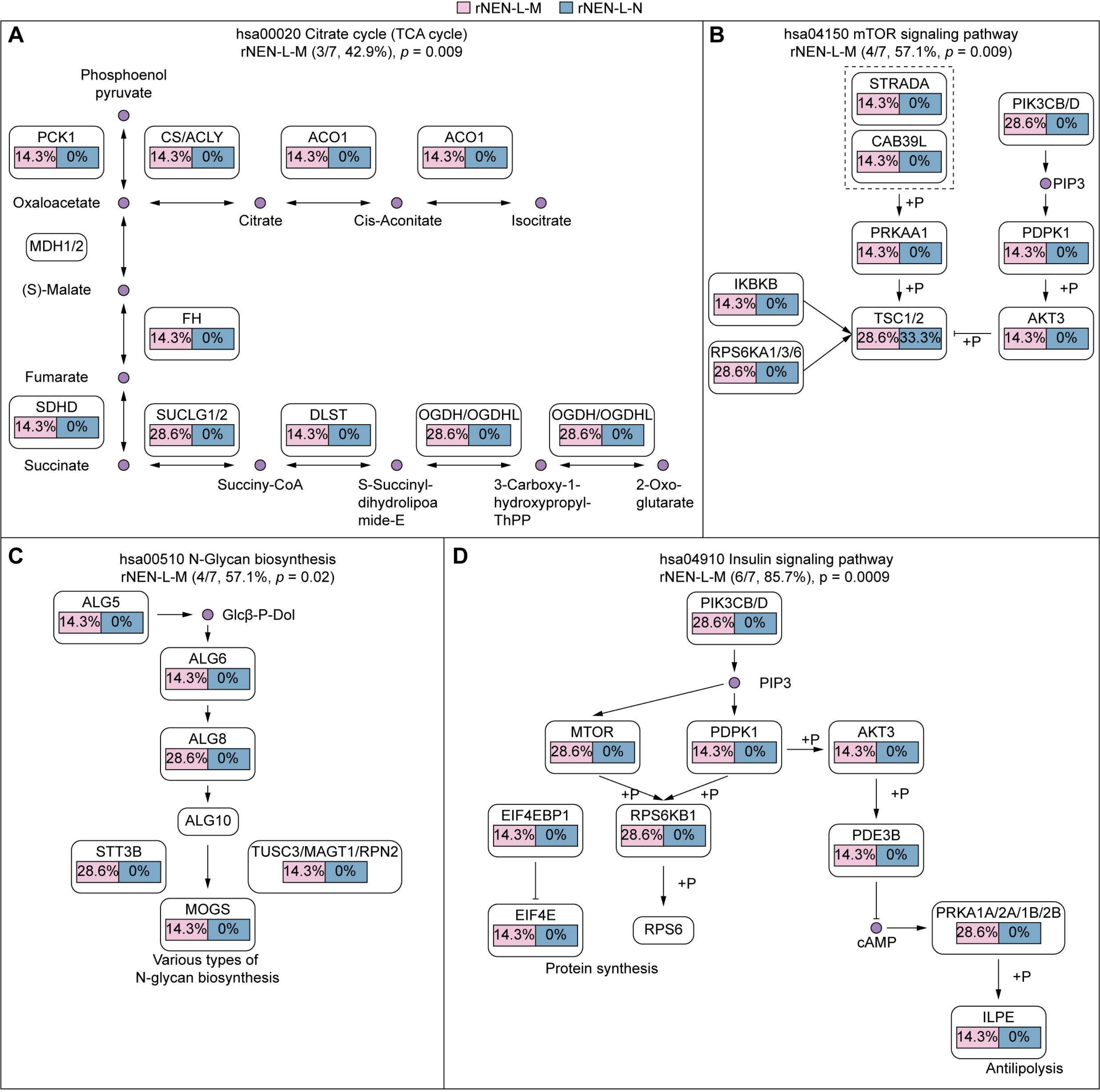

rNEN-L-M
rNEN-L-N
A
hsa00020 Citrate cycle (TCA cycle)
rNEN-L-M (3/7, 42.9%), p = 0.009
Phosphoenol pyruvate
PCK1 14.3% 0%
CS/ACLY 14.3% 0%
ACO1 14.3% 0%
ACO1 14.3% 0%
Oxaloacetate
Citrate
Cis-Aconitate
Isocitrate
MDH1/2
(S)-Malate
FH 14.3% 0%
Fumarate
SDHD 14.3% 0%
SUCLG1/2 28.6% 0%
DLST 14.3% 0%
OGDH/OGDHL 28.6% 0%
OGDH/OGDHL 28.6% 0%
Succinate
Succiny-CoA
S-Succinyl-dihydrolipoamide-E
3-Carboxy-1-hydroxypropyl-ThPP
2-Oxo-glutarate
B
hsa04150 mTOR signaling pathway
rNEN-L-M (4/7, 57.1%, p = 0.009)
STRADA 14.3% 0%
CAB39L 14.3% 0%
PIK3CB/D 28.6% 0%
PIP3
+P
PRKAA1 14.3% 0%
PDPK1 14.3% 0%
IKBKB 14.3% 0%
TSC1/2 28.6% 33.3%
AKT3 14.3% 0%
RPS6KA1/3/6 28.6% 0%
C
hsa00510 N-Glycan biosynthesis
rNEN-L-M (4/7, 57.1%, p = 0.02)
ALG5 14.3% 0%
Glcβ-P-Dol
ALG6 14.3% 0%
ALG8 28.6% 0%
ALG10
STT3B 28.6% 0%
TUSC3/MAGT1/RPN2 14.3% 0%
MOGS 14.3% 0%
Various types of N-glycan biosynthesis
D
hsa04910 Insulin signaling pathway
rNEN-L-M (6/7, 85.7%), p = 0.0009
PIK3CB/D 28.6% 0%
PIP3
MTOR 28.6% 0%
PDPK1 14.3% 0%
AKT3 14.3% 0%
EIF4EBP1 14.3% 0%
RPS6KB1 28.6% 0%
PDE3B 14.3% 0%
EIF4E 14.3% 0%
RPS6
cAMP
PRKA1A/2A/1B/2B 28.6% 0%
Protein synthesis
ILPE 14.3% 0%
Antilipolysis

Figure S4

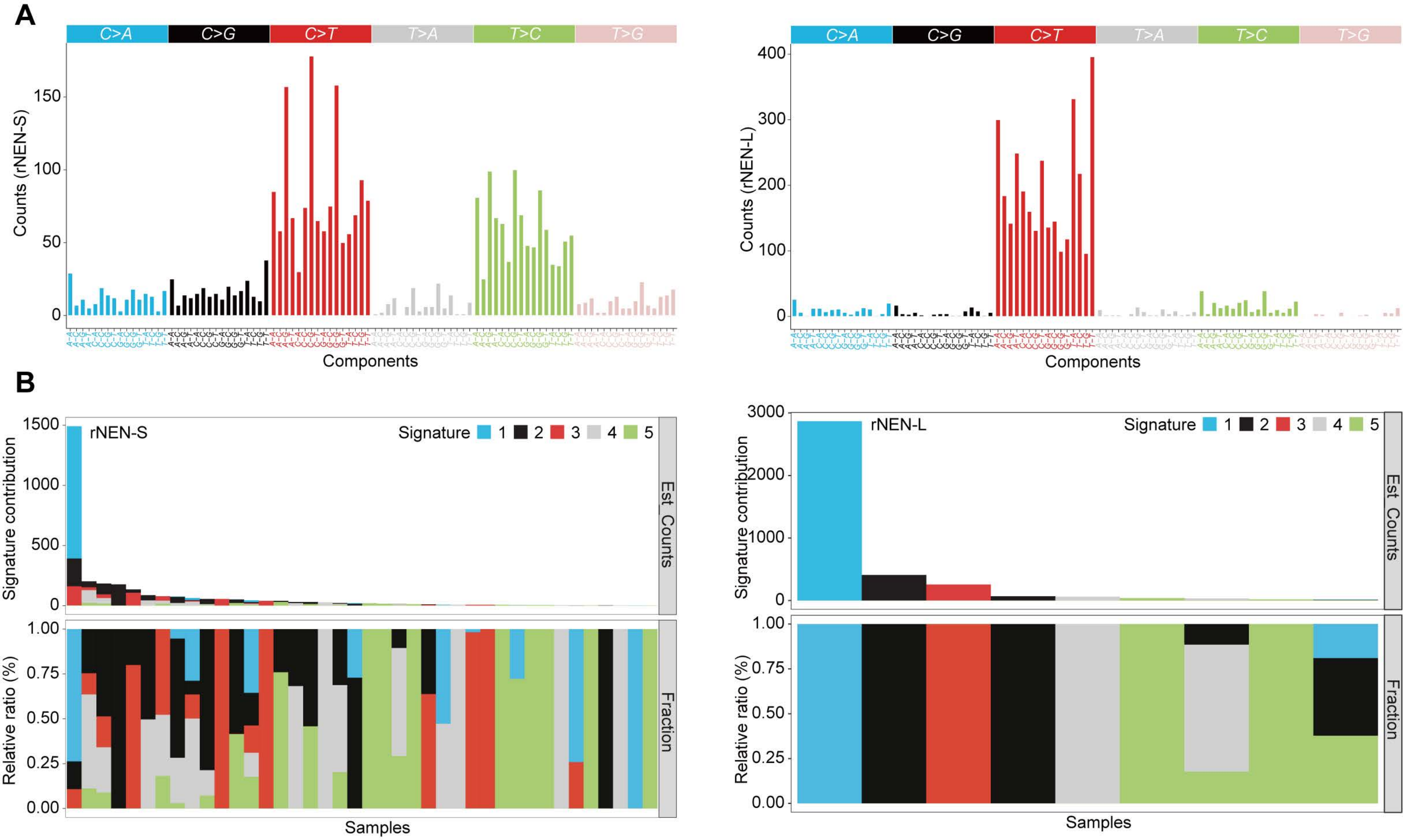
A
C>A
C>G
C>T
T>A
T>C
T>G
Counts (rNEN-S)
0
50
100
150
Components
Counts (rNEN-L)
0
100
200
300
400
Components
B
rNEN-S
Signature 1 2 3 4 5
Signature contribution
0
500
1000
1500
Est_Counts
Relative ratio (%)
0.00
0.25
0.50
0.75
1.00
Fraction
Samples
rNEN-L
Signature 1 2 3 4 5
Signature contribution
0
1000
2000
3000
Est_Counts
Relative ratio (%)
0.00
0.25
0.50
0.75
1.00
Fraction
Samples

Figure S5

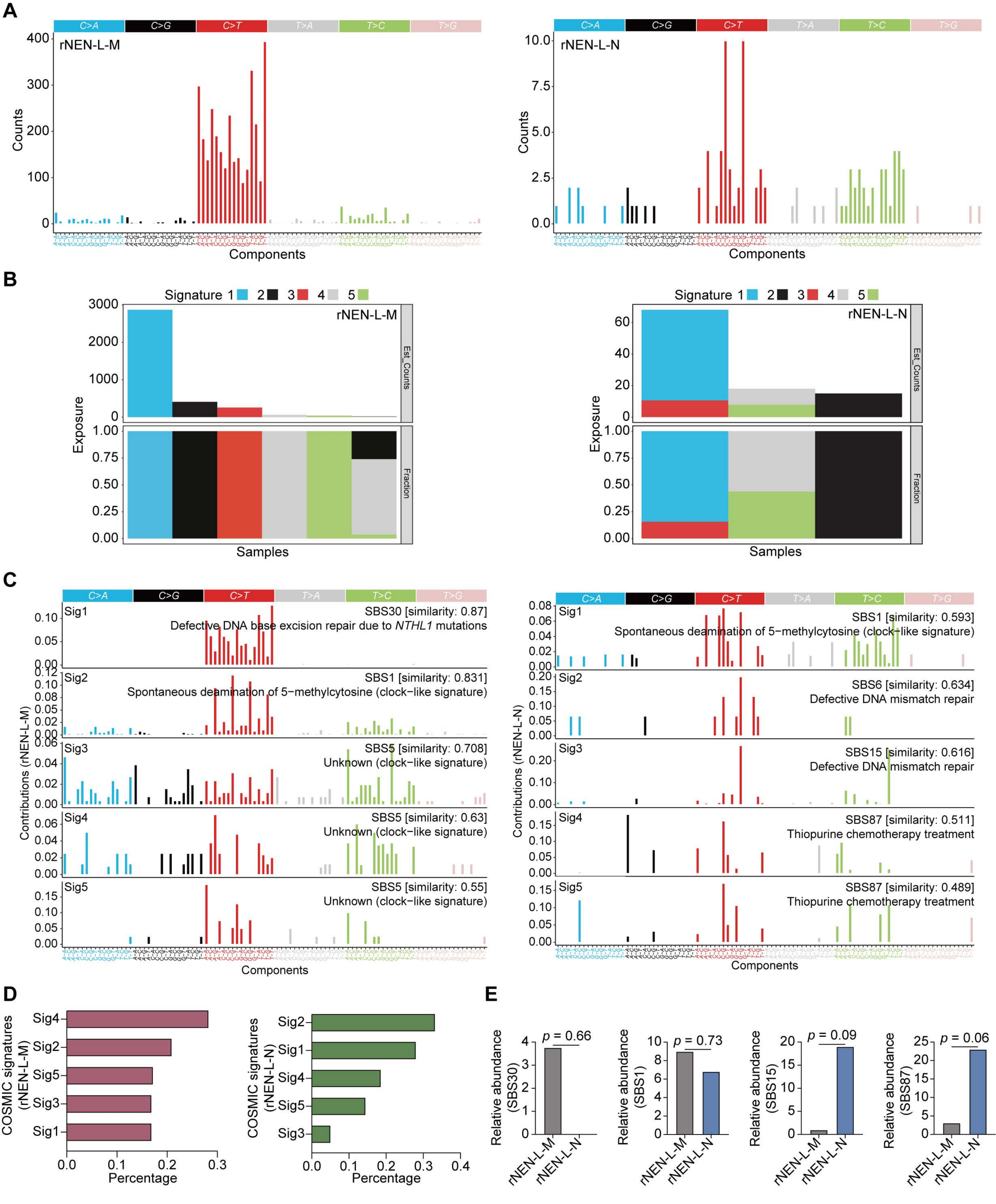
A
rNEN-L-M
rNEN-L-N
C>A
C>G
C>T
T>A
T>C
T>G
Counts
Components
B
Signature 1 2 3 4 5
Exposure
Est_Counts
Fraction
Samples
C
Sig1
SBS30 [similarity: 0.87]
Defective DNA base excision repair due to NTHL1 mutations
Sig2
SBS1 [similarity: 0.831]
Spontaneous deamination of 5−methylcytosine (clock−like signature)
Sig3
SBS5 [similarity: 0.708]
Unknown (clock−like signature)
Sig4
SBS5 [similarity: 0.63]
Unknown (clock−like signature)
Sig5
SBS5 [similarity: 0.55]
Unknown (clock−like signature)
Contributions (rNEN-L-M)
Sig1
SBS1 [similarity: 0.593]
Spontaneous deamination of 5−methylcytosine (clock−like signature)
Sig2
SBS6 [similarity: 0.634]
Defective DNA mismatch repair
Sig3
SBS15 [similarity: 0.616]
Defective DNA mismatch repair
Sig4
SBS87 [similarity: 0.511]
Thiopurine chemotherapy treatment
Sig5
SBS87 [similarity: 0.489]
Thiopurine chemotherapy treatment
Contributions (rNEN-L-N)
Components
D
COSMIC signatures (rNEN-L-M)
COSMIC signatures (rNEN-L-N)
Percentage
E
Relative abundance (SBS30)
p = 0.66
Relative abundance (SBS1)
p = 0.73
Relative abundance (SBS15)
p = 0.09
Relative abundance (SBS87)
p = 0.06
rNEN-L-M
rNEN-L-N

Figure S6

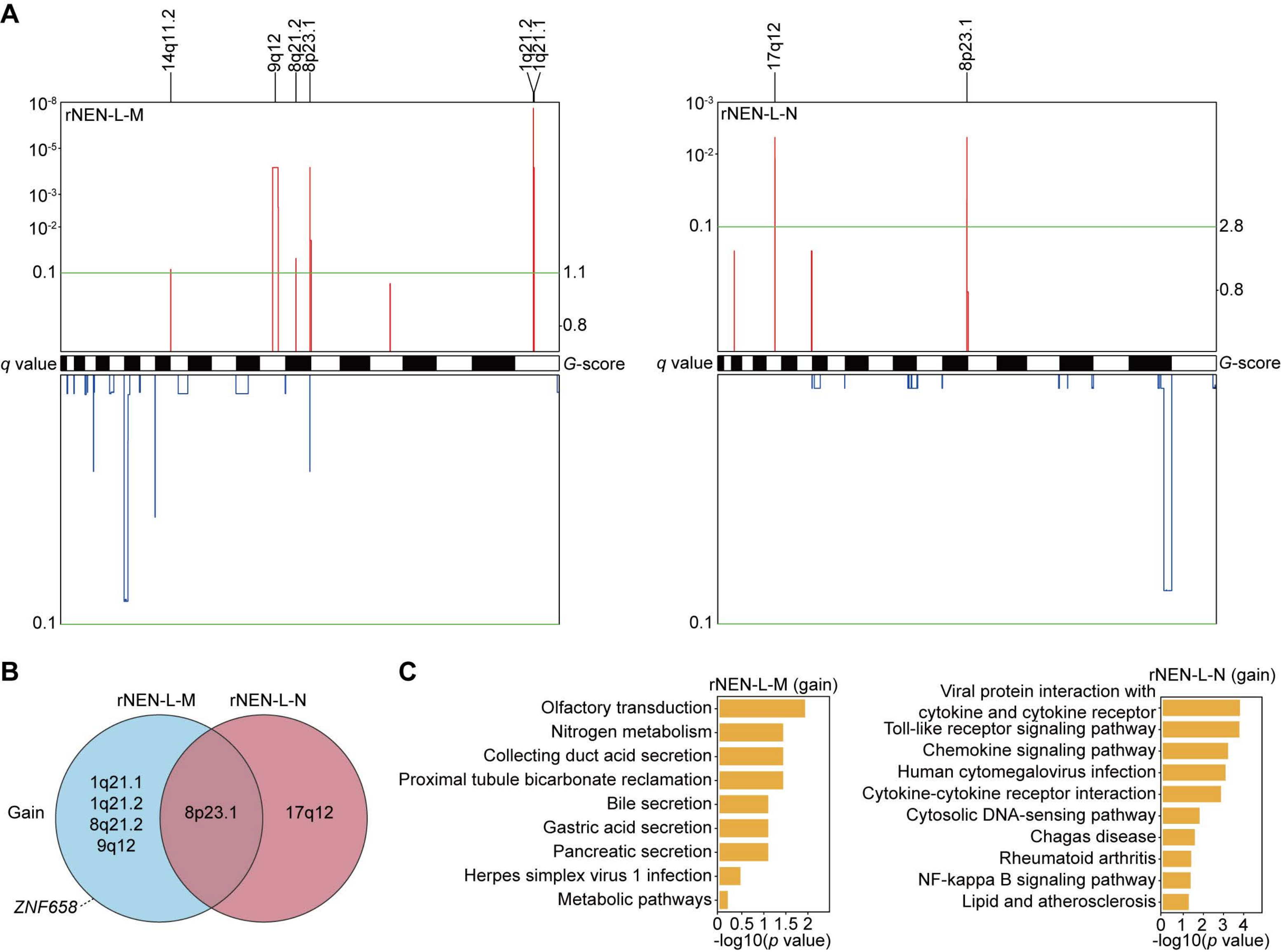
A
14q11.2
9q12
8q21.2
8p23.1
1q21.2
1q21.1
rNEN-L-M
q value
G-score
17q12
8p23.1
rNEN-L-N
B
rNEN-L-M
rNEN-L-N
Gain
1q21.1
1q21.2
8q21.2
9q12
8p23.1
17q12
ZNF658
C
rNEN-L-M (gain)
Olfactory transduction
Nitrogen metabolism
Collecting duct acid secretion
Proximal tubule bicarbonate reclamation
Bile secretion
Gastric acid secretion
Pancreatic secretion
Herpes simplex virus 1 infection
Metabolic pathways
-log10(p value)
rNEN-L-N (gain)
Viral protein interaction with cytokine and cytokine receptor
Toll-like receptor signaling pathway
Chemokine signaling pathway
Human cytomegalovirus infection
Cytokine-cytokine receptor interaction
Cytosolic DNA-sensing pathway
Chagas disease
Rheumatoid arthritis
NF-kappa B signaling pathway
Lipid and atherosclerosis
-log10(p value)

Figure S7

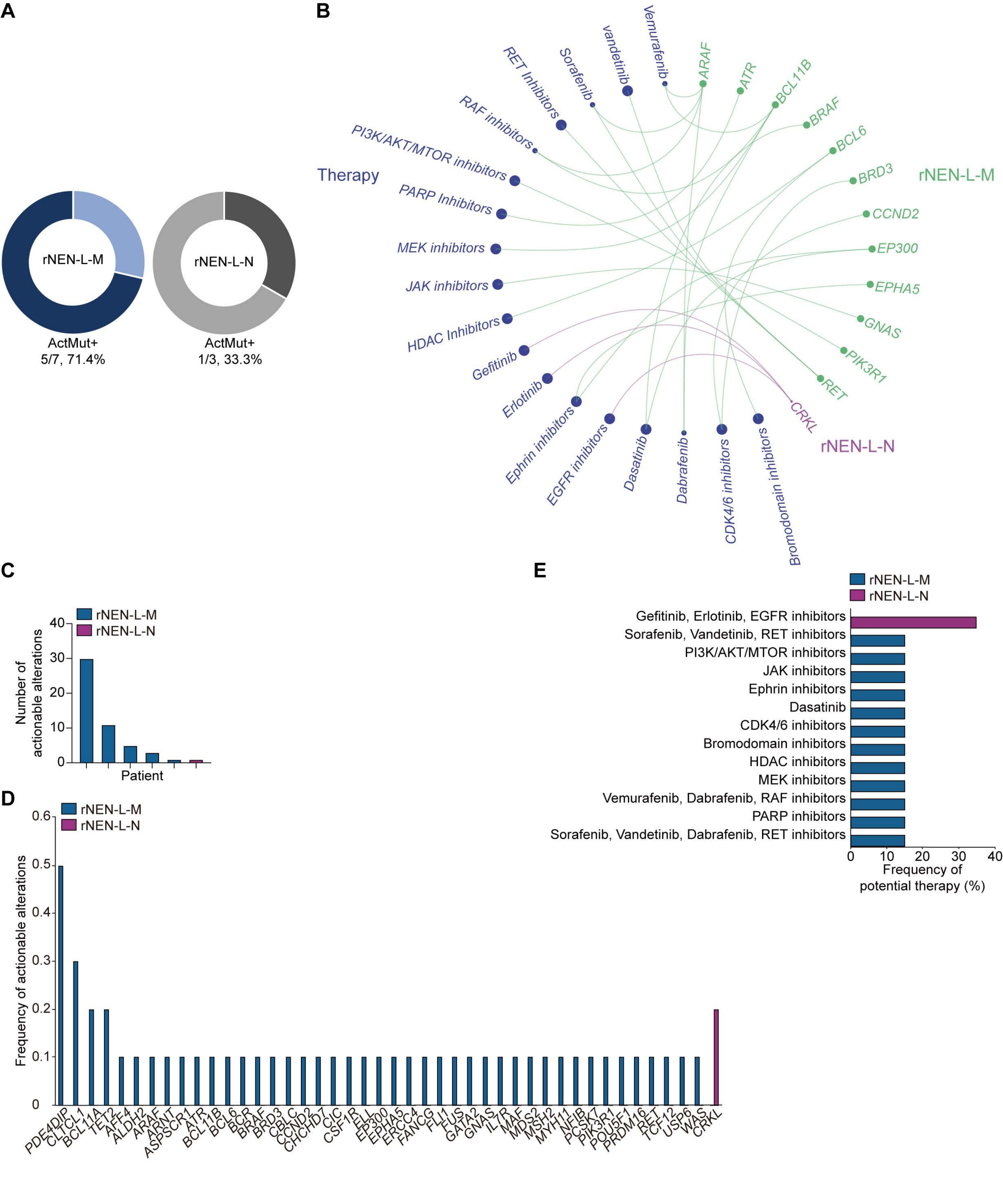
A
rNEN-L-M
rNEN-L-N
ActMut+
5/7, 71.4%
ActMut+
1/3, 33.3%
B
Therapy
rNEN-L-M
rNEN-L-N
Vemurafenib
vandetinib
Sorafenib
RET Inhibitors
RAF inhibitors
PI3K/AKT/MTOR inhibitors
PARP Inhibitors
MEK inhibitors
JAK inhibitors
HDAC Inhibitors
Gefitinib
Erlotinib
Ephrin inhibitors
EGFR inhibitors
Dasatinib
Dabrafenib
CDK4/6 inhibitors
Bromodomain inhibitors
ARAF
ATR
BCL11B
BRAF
BCL6
BRD3
CCND2
EP300
EPHA5
GNAS
PIK3R1
RET
CRKL
C
rNEN-L-M
rNEN-L-N
Number of actionable alterations
Patient
D
rNEN-L-M
rNEN-L-N
Frequency of actionable alterations
PDE4DIP
CLTCL1
BCL11A
TET2
AFF4
ALDH2
ARAF
ARNT
ASPSCR1
ATR
BCL11B
BCL6
BCR
BRAF
BRD3
CBLC
CCND2
CHCHD7
CIC
CSF1R
ELL
EP300
EPHA5
ERCC4
FANCG
FLI1
FUS
GATA2
GNAS
IL7R
MAF
MDS2
MSH2
MYH11
NFIB
PCSK7
PIK3R1
POU5F1
PRDM16
RET
TCF12
USP6
WAS
CRKL
E
rNEN-L-M
rNEN-L-N
Gefitinib, Erlotinib, EGFR inhibitors
Sorafenib, Vandetinib, RET inhibitors
PI3K/AKT/MTOR inhibitors
JAK inhibitors
Ephrin inhibitors
Dasatinib
CDK4/6 inhibitors
Bromodomain inhibitors
HDAC inhibitors
MEK inhibitors
Vemurafenib, Dabrafenib, RAF inhibitors
PARP inhibitors
Sorafenib, Vandetinib, Dabrafenib, RET inhibitors
Frequency of potential therapy (%)

Figure S8
